# CRISPR-Cas9 Based Genome Editing Reveals New Insights into MicroRNA Function and Regulation in Rice

**DOI:** 10.3389/fpls.2017.01598

**Published:** 2017-09-13

**Authors:** Jianping Zhou, Kejun Deng, Yan Cheng, Zhaohui Zhong, Li Tian, Xu Tang, Aiting Tang, Xuelian Zheng, Tao Zhang, Yiping Qi, Yong Zhang

**Affiliations:** ^1^Department of Biotechnology, School of Life Science and Technology, Center for Informational Biology, University of Electronic Science and Technology of China Chengdu, China; ^2^Jiangsu Key Laboratory of Crop Genetics and Physiology, Co-Innovation Center for Modern Production Technology of Grain Crops, Key Laboratory of Plant Functional Genomics of the Ministry of Education, Yangzhou University Yangzhou, China; ^3^Department of Plant Science and Landscape Architecture, University of Maryland College Park, MD, United States; ^4^Institute for Bioscience and Biotechnology Research, University of Maryland Rockville, MD, United States

**Keywords:** microRNAs, CRISPR-Cas9, genome editing, rice, *OsMIR408*, *OsMIR528*

## Abstract

MicroRNAs (miRNAs) are small non-coding RNAs that play important roles in plant development and stress responses. Loss-of-function analysis of miRNA genes has been traditionally challenging due to lack of appropriate knockout tools. In this study, single miRNA genes (OsMIR408 and OsMIR528) and miRNA gene families (miR815a/b/c and miR820a/b/c) in rice were targeted by CRISPR-Cas9. We showed single strand conformation polymorphism (SSCP) is a more reliable method than restriction fragment length polymorphism (RFLP) for identifying CRISPR-Cas9 generated mutants. Frequencies of targeted mutagenesis among regenerated T0 lines ranged from 48 to 89% at all tested miRNA target sites. In the case of miRNA528, three independent guide RNAs (gRNAs) all generated biallelic mutations among confirmed mutant lines. When targeted by two gRNAs, miRNA genes were readily to be deleted at a frequency up to 60% in T0 rice lines. Thus, we demonstrate CRISPR-Cas9 is an effective tool for knocking out plant miRNAs. Single-base pair (bp) insertion/deletion mutations (indels) in mature miRNA regions can lead to the generation of functionally redundant miRNAs. Large deletions at either the mature miRNA or the complementary miRNA^*^ were found to readily abolish miRNA function. Utilizing mutants of *OsMIR408* and *OsMIR528*, we find that knocking out a single miRNA can result in expression profile changes of many other seemingly unrelated miRNAs. In a case study on *OsMIR528*, we reveal it is a positive regulator in salt stress. Our work not only provides empirical guidelines on targeting miRNAs with CRISPR-Cas9, but also brings new insights into miRNA function and complex cross-regulation in rice.

## Introduction

**Figure 1:**
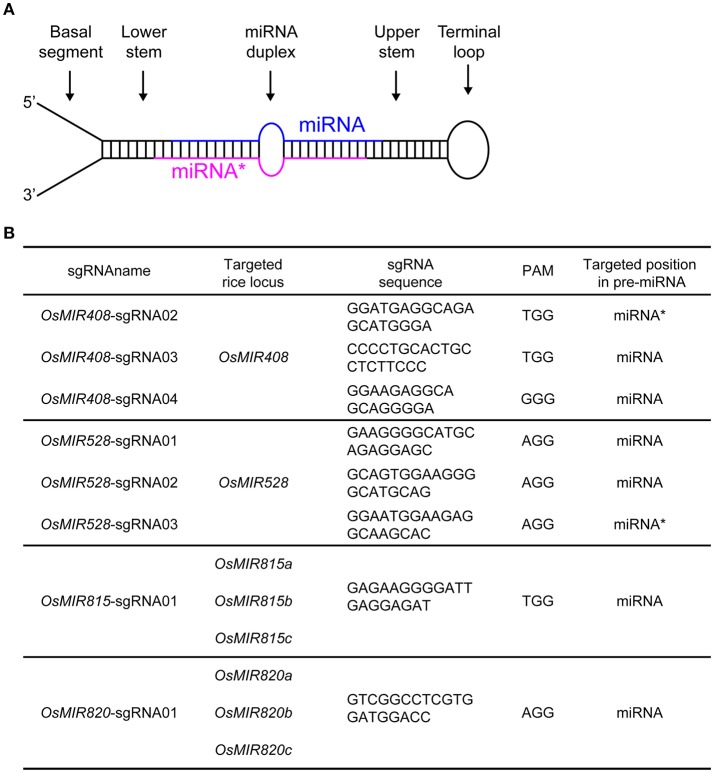
Targeting rice endogenous microRNA genes with CRISPR-Cas9. **(A)** Illustration of a primary microRNA (pri-miRNA) transcript which forms a stem loop structure. The mature miRNA and its complementary strand (miRNA^*^) are color-coded in blue and red, respectively. **(B)** Summary of sgRNAs and their targeting miRNAs in rice.

MicroRNAs (miRNAs) are small non-coding RNAs that function as important post-transcriptional regulators in plants and animals. Biogenesis of miRNAs starts with processing of RNA Polymerase II promoter driven primary miRNA (pri-miRNA) transcripts that fold back to form hairpin structures (Bartel, 2004; Voinnet, 2009). After processing, mature miRNAs are bound to Argonaute (Ago) proteins forming miRNA/Ago complexes which then search for cognate messenger RNAs (mRNA) targeting them for degradation and/or translational silencing (Tolia and Joshua-Tor, 2007; Chen, 2009; Meister, 2013). MiRNA biogenesis starts with the transcription of long pri-miRNAs by RNA polymerase II. Each pri-miRNA is characterized by a stem-loop structure containing a duplex of miRNA and its complementary strand (miRNA^*^) (Zhu et al., 2013; Figure 1A). In plants, miRNAs recognize target mRNAs by highly specific complementary base-pairing mechanisms and regulate many processes, such as development and responses to biotic and abiotic stress (Chen, 2012; Kumar, 2014). For example, a previous study showed that miR528 can positively affect plant response to salinity stress through down regulation of its target genes, such as *ascorbic acid oxidase* (*AAO*) and *copper ion binding protein 1* (*CBP1*) in creeping bentgrass (Yuan et al., 2015). Recently, miR528 was shown to negatively regulate viral resistance in rice by cleaving *L-ascorbate oxidase* (*AO*) messenger RNA, thereby reducing AO-mediated accumulation of reactive oxygen species (Wu et al., 2017). MiR408 targets Cu2+-containing plantacyanin, laccase, and *P*-type ATPases genes in Arabidopsis (Ma C. et al., 2015). When copper is deficient, the transcription factor SPL7 activates the expression of miR408, miR398, and miR397 to repress their targets in Arabidopsis (Yamasaki et al., 2009). Overexpression of miR408 resulted in altered abiotic stress responses in Arabidopsis and chickpea (Hajyzadeh et al., 2015; Ma C. et al., 2015).

Genome editing with sequence specific nucleases (SSNs) is a powerful genetic engineering approach in which DNA is deleted, inserted or replaced at endogenous loci in a given genome (Voytas, 2013). SSNs function as molecular scissors to induce double strand breaks (DSBs) on target DNA at a predefined locus. The formation of DSBs trigger endogenous DNA repair pathways in cells. The dominant DNA repair pathway in higher eukaryotes is non-homologous end joining (NHEJ), which is relatively error-prone and can lead to insertions or deletions (indels) or mismatch mutations at target loci. Alternatively, homology-directed repair (HDR) requires a DNA donor template and is less efficient compared to NHEJ. In recent years, genome editing has emerged from the rapid development of SSN platforms such as zinc finger nuclease (ZFN) (Zhang et al., 2010; Sander et al., 2011), transcriptional activator-like effector nuclease (TALEN) (Christian et al., 2010; Cermak et al., 2011; Shan et al., 2013a; Zhang et al., 2013), clustered regularly interspaced short palindromic repeats and associated protein 9 (CRISPR-Cas9) (Jinek et al., 2012; Cong et al., 2013; Feng et al., 2013; Shan et al., 2013b) and CRISPR-Cpf1 (Tang et al., 2017). CRISPR-Cas9 is highly facile as it targets DNA using a single guide RNA (sgRNA) through Watson-Crick base pairing. This nucleotide based targeting mechanism has made CRISPR-Cas9 the preferred SSN for genome editing across organisms. Since its first demonstration in editing plant genomes in 2013 (Li et al., 2013; Nekrasov et al., 2013; Shan et al., 2013b), CRISPR-Cas9 has been constantly improved and widely adopted for editing genomes of many plant species (Paul and Qi, 2016; Demirci et al., 2017).

Post-transcriptional technologies that sequester miRNAs (Franco-Zorrilla et al., 2007; Todesco et al., 2010) or degrade miRN*As* (Yan et al., 2012) have been used for loss-of-function analysis in plants. However, these methods produced variable results in miRNA inhibition when tested in plants (Reichel et al., 2015). Using CRISPR-Cas9, it is now feasible to directly generate miRNA knockout mutants, which are superior for carrying out reverse genetic research strategies. Recently, two soybean miRNA genes, miR1514, and miR1509 (Jacobs et al., 2015) and two Arabidopsis miRNA genes, miR169a, and miR827a (Zhao et al., 2016), were targeted via the CRISPR/Cas9 system. To date however, a demonstration of CRISPR-Cas9 targeting miRNA genes for obtaining mutant plants has not yet been reported in major crops. Using rice as a crop model, we tested this application and explored the following technical and biological questions: (1) What are the effects of different mutations on the production of mature miRNAs and their function? (2) What would be the most effective way for generating *miRNA* null alleles? (3) What would be a good genotyping method for identifying *miRNA* mutants prior to Sanger sequencing? (4) What are the phenotypic and molecular consequences of knocking out a *miRNA* gene? To answer these questions, we applied CRISPR-Cas9 to target multiple *miRNA* and *miRNA* family genes in rice. Our results provide new technical and biological insights into reverse genetic studies of *miRNA* genes using CRISPR-Cas9 in rice.

## Materials and methods

### Plant material and growth condition

The rice cultivar Nipponbare (*Oryza sativa* L. japonica) was used as the WT control and transformation host. Mature seeds were geminated for 2 days at 37°C in the dark. Germinated seeds were then transferred to soil, and seedlings were grown under standard greenhouse conditions (16-h light at 30°C/8-h night at 22°C).

### Vector construction

Cas9 expression backbone vector pZHY988 (Tang et al., 2016) was used in this paper. For our single-target vector, the individual annealed sgRNA oligonucleotide pair (Supplementary Table 1) was cloned into the region between the *OsU6* promoter and the sgRNA scaffold. For the dual-target vector, two expression cassettes of *OsU6* promoter-gRNA scaffolds-OsU6 terminator were cloned into pZHY988 using fusion PCR and ligase. And the primers were listed in Supplementary Table 1.

### Rice protoplast transformation and stable transformation

The japonica cultivar Nipponbare was used in this experiment. Isolation and transformation of rice protoplasts were carried out as previously described (Tang et al., 2016). The rice calli were cultured from mature embryo and Agrobacterium mediated transformation of T-DNA vectors into rice calli was conducted according to an established protocol (Toki et al., 2006).

### Genotyping of genome editing events

Total genomic DNA was extracted using the cetyltrimethyl ammonium bromide (CTAB) method (Stewart and Via, 1993). PCR was performed to amplify the genomic regions surrounding the CRISPR-Cas9 target sites using the specific primers (Supplementary Table 1). The PCR fragments for OsMIR528-sgRNA02 were digested with the restriction enzyme SphI, in RFLP analysis. For SSCP analysis, PCR products from the targeted regions were first analyzed using PAGE assay (Zheng et al., 2016) and positive samples were subsequently subjected to Sanger sequencing. Sequencing results containing biallelic mutations were decoded with an establish tool (Ma X. et al., 2015).

### Small RNA sequencing

The biallelic T1 mutants of OsMIR408-sgRNA03-19 (−13/−8 bp), OsMIR528-sgRNA01-15 (−54/−4 bp), and OsMIR528-sgRNA-02-08 (−3/+1 bp) with no vector and WT were chosen for small RNA sequencing. Total RNA of 10 whole plants for each sample was isolated using TRIzol (Invitrogen, USA) at three leaves stage. Library construction, small RNA sequencing using Illumina Hiseq 2500 planform and data processing and analysis were carried out at Biomarker Technologies Co., Ltd. The clean sRNAs were mapped to GenBank (https://www.ncbi.nlm.nih.gov/), Rfam database (http://rfam.xfam.org/), and rRNA, tRNA, snRNA, and snoRNA were discarded from the sRNA reads using bowtie2 software (Langmead et al., 2009). The unannotated sequences were then analyzed by miRDeep2 software package (Friedlander et al., 2012) to predict miRNAs according to rice gemome database (ftp://ftp.ensemblgenomes.org/pub/plants/release-24/fasta/oryza_sativa/). Differentially expressed miRNAs were identified using the TPM (Fahlgren et al., 2007) and IDEG6 (Romualdi et al., 2003). And the small RNA sequences have been deposited into NCBI SRA database under accession number: SRS2475225.

### RNA extraction and qRT-PCR

The genotype of independent seedlings was identified through SSCP and sequencing before RNA extraction. Total RNA was extracted using TRIzol, treated with DNase I and then used for cDNA synthesis. For miRNA detection, miRNA cDNA synthesis was carried out using the miRcute miRNA cDNA kit (Tiangen, China) per the manufacturer's instructions. SYBR green-based qRT-PCR was performed using a specific forward primer (Supplementary Table 1) and a universal reverse primer provided by the kit (Tiangen, China). Actin mRNA was used as an internal control. For qRT-PCR of mRNA, reverse transcription (RT) was carried out using RevertAid First Strand cDNA Synthesis Kit (Thermo Scientific, USA), and qRT-PCR was performed with SuperReal PreMix Plus (SYBR Green) PCR master mix kit (Tiangen, China) per the manufacturer's instructions. Actin mRNA was used as an internal control. The relative levels of gene expression were calculated using the 2-ΔΔ cycle threshold (CT) method. Three biological replicates (three independent T1 seedlings with the same genotype and without Cas9 vector for each salt treated sample) were examined to ensure reproducibility. The experiments were performed three times independently with similar results.

### Salt stress treatment and chlorophyll content analysis

Mature seeds were geminated for 2 days at 37°C in the dark, the germinated seeds were planted into containers supplemented with water-soluble fertilizer in the growth chamber under long-day conditions (16 h light at 28°C and 8 h dark at 22°C). After 14 days, 60 mM NaCl solution along with water-soluble fertilizer was added to the containers. After 7 days' salt treatment, the seedlings were harvested for further analysis. The genotype of independent seedling was identified through SSCP and sequencing. Chlorophyll was extracted and measured according to a previously described protocol (Wang et al., 2008). Briefly, leaf tissue under salt stress were homogenized in 80% acetone at 4°C, the homogenates centrifuged and fluorescence measured at 662, 645, and 440 nm with Fluorescence Spectrometer HITACHI U2910 (JAPAN). Leaf Chl contents were estimated according to Wang et al. (2008).

## Results

### High efficiency rice miRNA mutagenesis by CRISPR-Cas9

We decided to target miRNA: miRNA^*^ duplex regions with CRISPR-Cas9 as mutations in these regions are likely to impact miRNA biogenesis and function. We chose to work on *OsMIR408, OsMIR528, OsMIR815*, and *OsMIR820* as these miRNAs have not been genetically studied in rice. *OsMIR408* and *OsMIR528* were each targeted by three sgRNAs (Figure 1B). Since *OsMIR815* and *OsMIR820* each contain three homologous members, one sgRNA was designed for targeting the entire family based on sequence homology (Figure 1B). A total of 8 sgRNAs (Supplementary Figure 1) were cloned into CRISPR-Cas9 T-DNA vectors, in which Cas9 was expressed under a maize ubiquitin promoter and the sgRNAs were expressed under the *OsU6* promoter. We first tested six such T-DNA vectors in a rice protoplast system. As we limited the sgRNA targeting site to the miRNA or miRNA^*^ region, it was difficult to find acceptable restriction enzyme sites for restriction fragment length polymorphism (RFLP) based mutation analysis (Supplementary Figure 2A). Rather, direct Sanger sequencing of PCR products was used, which revealed mutations at target sites (Supplementary Figure 2B).

**Figure 2:**
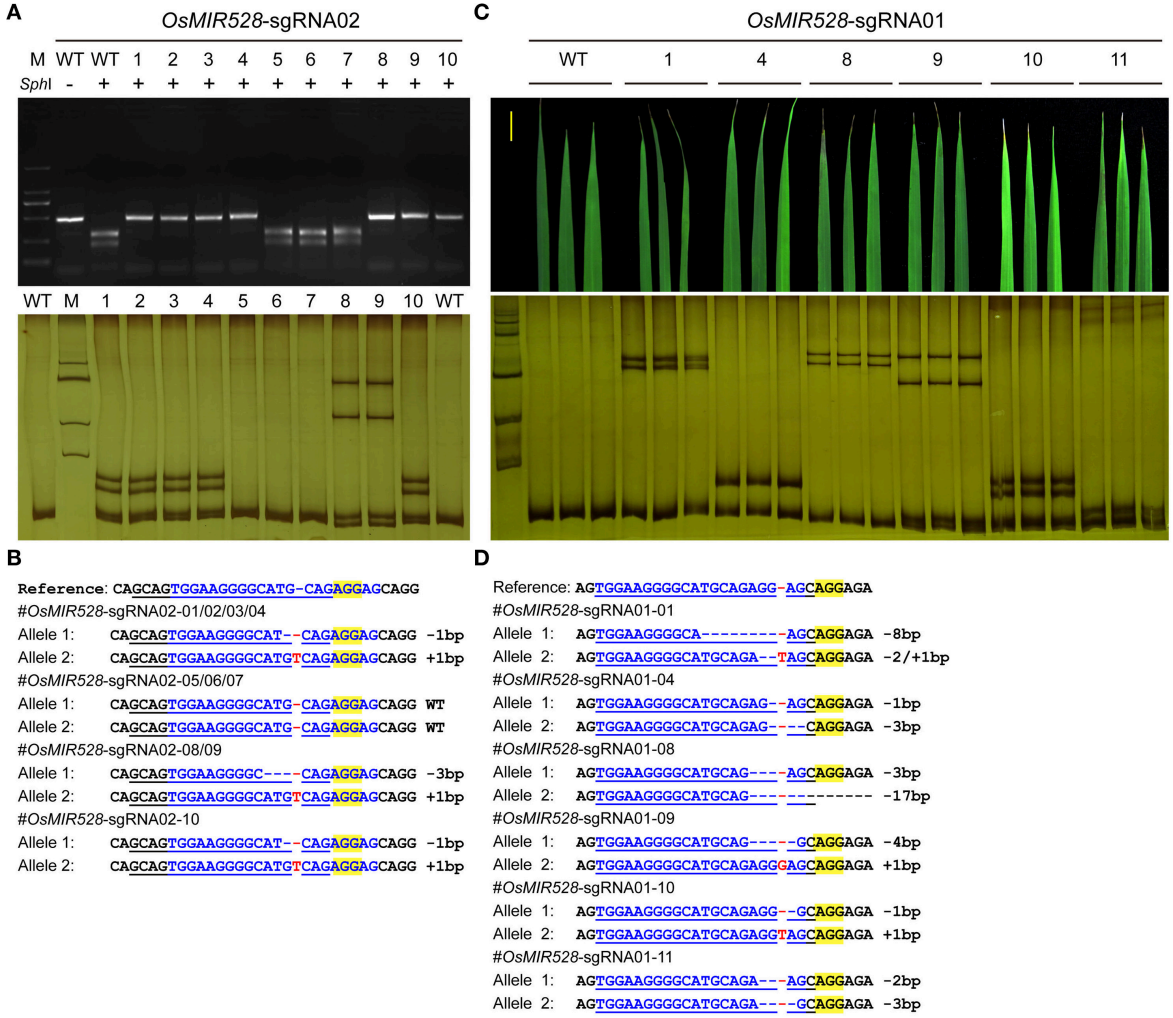
SSCP is superior to RFLP on genotyping mutations at microRNA loci. **(A)** RFLP (upper panel) and SSCP (lower panel) analysis of 10 independent *OsMIR528-sgRNA02* T0 lines. PCR products were digested by *SphI* in the RFLP analysis. **(B)** Sanger sequencing of the target site in the *OsMIR528-sgRNA02* T0 lines. **(C)** SSCP analysis of 6 independent *OsMIR528-sgRNA01* T0 lines. Three independent leaves of each T0 plant were genotyped. **(D)** Sanger Sequencing of the target site in the *OsMIR528-sgRNA01* T0 lines.

We then pursued stable transgenic T0 lines with the Agrobacterium mediated transformation of rice calli. To screen mutants generated by *OsMIR528-sgRNA02*, we compared RFLP with single strand conformation polymorphism (SSCP), which is a method we recently applied for genotyping CRISPR-Cas9 induced mutations (Zheng et al., 2016). Although RFLP could identify mutants (Figure 2A, upper panel), SSCP showed higher resolution in distinguishing different mutations (Figure 2A, lower panel). Among 10 T0 lines, 7 carried biallelic mutations at the target site (Table 1, Figure 2B). Due to its effectiveness, we applied SSCP for genotyping all T0 lines with the remaining CRISPR-Cas9 constructs. For *OsMIR528-sgRNA01* T0 lines, 26 out of 34 contain mutations, all of which are biallelic (Table 1, Supplementary Figure 3). Three independent leaves from individual T0 lines were analyzed by SSCP and the results suggest each plant carried homogenous mutations (Figures 2C,D), suggesting these mutations should be readily transmitted into the next generation. We followed 7 biallelic lines to the T1 generation and found segregation patterns all followed Mendelian inheritance (Supplementary Table 2). For *OsMIR528-sgRNA03* T0 lines, 15 out of 18 were identified as mutants by SSCP (Supplementary Figure 4). Sanger sequencing further confirmed these mutants and revealed one additional homozygous mutant (+1/+1 bp) that was missed by the SSCP assay (Table 1, Supplementary Figure 5). For *OsMIR408-sgRNA03* T0 lines, 10 out of 21 were identified as mutants by SSCP (Supplementary Figure 6A), which were later confirmed by Sanger sequencing (Supplementary Figure 6B). Among these mutants, 8 were biallelic and 2 were heterozygous (Table 1, Supplementary Figure 6B). Taken together, these results suggest targeted mutagenesis of *miRNA* genes in rice by CRISPR-Cas9 is very efficient and SSCP is a reliable genotyping method for mutant identification.

**Table 1:** Summary of mutation frequencies at targeted miRNA loci in T0 lines.

**Targeted rice gene**	**Transgenetic CRISPR/Cas9 vector**	**Tested T0 seedling**	**Mutated T0 seedling: number, ratio**	**Biallelic mutation: number, ratio**
*OsMIR408*	pZmUbi1::Cas9+OsU6::*OsMIR408−sgRNA03*	21	10, 47.6%	8, 80.0%
*OsMIR528*	pZmUbi1::Cas9+OsU6::*OsMIR528−sgRNA01*	34	26, 76.5%	26, 100%
	pZmUbi1::Cas9+OsU6::*OsMIR528−sgRNA02*	10	7, 70.0%	7, 100%
	pZmUbi1::Cas9+OsU6::*OsMIR528−sgRNA03*	18	16, 88.9%	16, 100%
*OsMIR815* (a/b/c)	pZmUbi1::Cas9+OsU6::*OsMIR815−sgRNA01*	20	15, 75.0%	*OsMIR815a*: 10, 66.7%
				*OsMIR815b*: 7, 46.7%
				*OsMIR815c*: 3, 20.0%
*OsMIR820* (a/b/c)	pZmUbi1::Cas9+OsU6::*OsMIR820−sgRNA01*	18	14, 77.8.0%	*OsMIR820a*: 10, 71.4%
				*OsMIR820b*: 13, 92.9%
				*OsMIR820c*: 10, 71.4%

### Simultaneous targeting of homologous miRNAs with a single sgRNA

We next targeted *miRNA* family members, *OsMIR815a/b/c* or *OsMIR820a/b/c*, with one sgRNA that recognizes all three family members. For *OsMIR815-sgRNA01*, 20 T0 lines were screened for mutations at *OsMIR815a, OsMIR815b*, and *OsMIR815c* loci by SSCP and followed with Sanger sequencing. SSCP was used to identify mutants at *OsMIR815a* and *OsMIR815c*, but not at *OsMIR815b* (Supplementary Figure 7). Sanger sequencing confirmed 15 lines containing mutations at the target loci (Supplementary Table 3). Among them, 10, 7, and 3 lines carried biallelic mutations at *OsMIR815a, OsMIR815b*, and *OsMIR815c*, respectively (Table 1, Supplementary Figure 8). Importantly, line # 01, 05 are triple biallelic mutants, which should have abolished the function of the entire miRNA family. For *OsMIR820-sgRNA01*, 18 T0 lines were screened directly with Sanger sequencing and 14 of them carried mutations (Supplementary Table 3). Among these mutants, many lines contained biallelic mutations at *OsMIR820a, OsMIR820b* and *OsMIR820c*, respectively (Table 1 and Supplementary Figures 9, 10). Significantly, 11 lines are triple biallelic mutants. The results suggest *miRNA* gene families can be effectively and simultaneously mutated by CRISPR-Cas9 in rice.

### Off-target analysis of miRNA-targeting sgRNAs

In our design, Cas9 target sites largely overlap with the DNA regions corresponding to mature miRNA or miRNA^*^ (Figure 1A). As miRNAs recognize target mRNAs by near perfect RNA: RNA base-pairing, it might be possible that these sgRNAs can target genes of miRNAs along with various off-target homologous genomic sequences. To investigate this off-targeting possibility, we examined 6 sgRNAs used in this study and chose 5 high probability off-target sites for each sgRNA assay. By randomly selecting five corresponding T0 plants for each sgRNA and surveying these potential off-targeting sites using Sanger sequencing of PCR products, we didn't find any mutation across all 30 potential off-target sites (Supplementary Table 4). Although we couldn't extensively look at all potentially off-target sites, the results suggest off-targeting effects from these sgRNAs are of less concern.

### Deletion of miRNA genes with two DSBs

**Figure 3:**
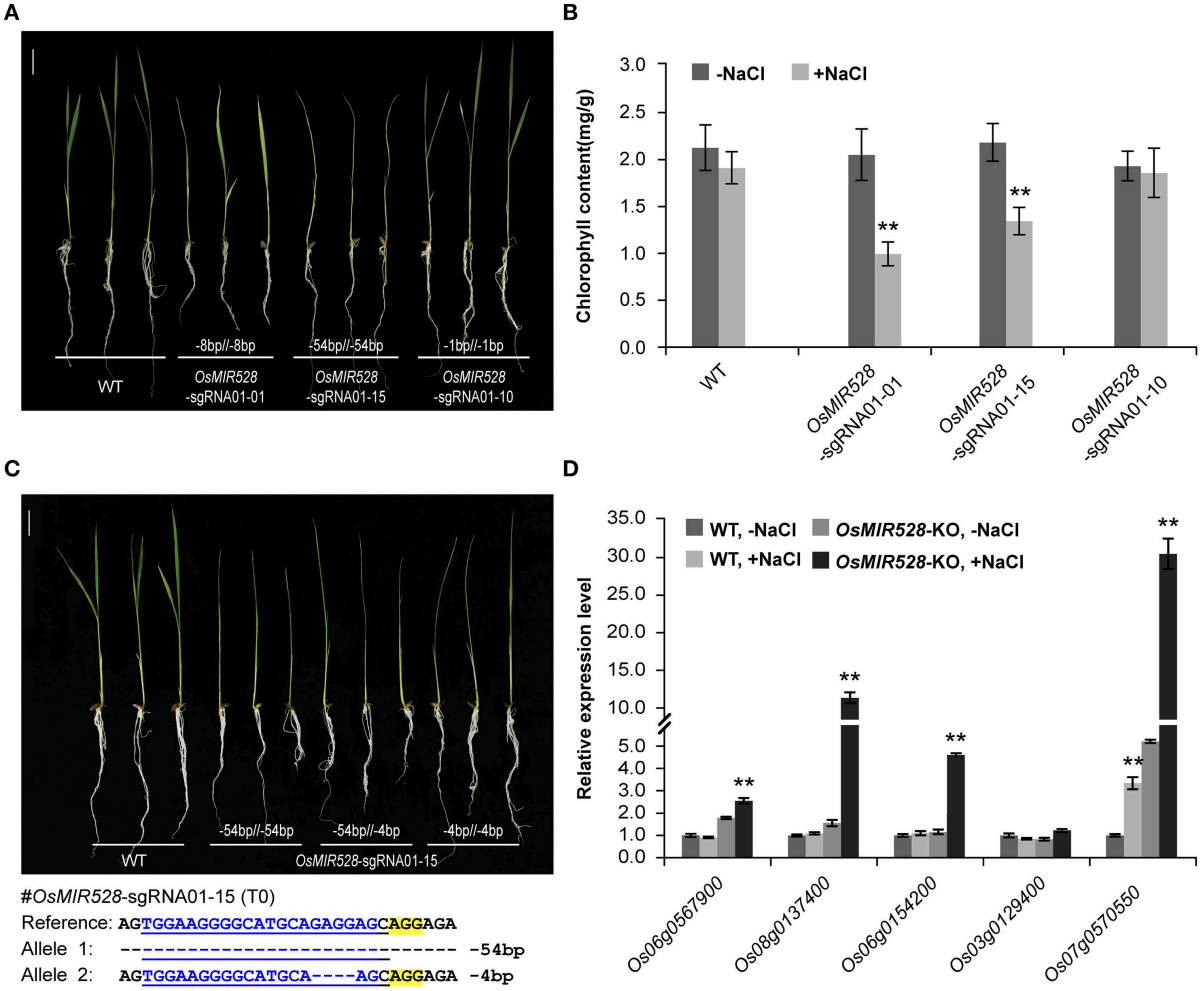
Loss of OsMIR528 function results in reduced tolerance to salt stress. **(A)** Phenotypic analysis of *OsMIR528* T1 mutant lines under salt stress. **(B)** Quantification of chlorophyll in OsMIR528 T1 mutant lines. **(C)** Large deletions lead to loss of function of OsMIR528. **(D)** Reactivation of OsMIR528's endogenous target genes in OsMIR528 knockout lines. Asterisks indicate significant differences according to Student's *t*-test; ^**^P ≤ 0.01.

After establishing an effective CRISPR-Cas9 based mutagenesis platform for *miRNAs*, we investigated what kind of mutations can result in loss-of-function. We focused our analysis on *OsMIR528* because prior studies suggest it may be involved in rice stress response (Cheah et al., 2015; Sharma et al., 2015). T1 plants from three independent T0 biallelic mutants of *OsMIR528-sgRNA01* were treated with salt stress by growing the seedlings in medium supplemented with 60 mM NaCl. Compared to the wild type (WT), two mutants showed delayed development (e.g., branching) and chlorosis phenotype (Figure 3A and Supplementary Figure 11), with the latter being further confirmed by quantification of chlorophyll (Figure 3B). The data provided genetic evidence that *OsMIR528* is positively involved in salt stress response. Interestingly, the *OsMIR528-sgRNA01-10* mutant with a single bp indel mutation emulated WT under salt stress (Figures 3A,B). These findings suggest that larger deletions, not 1-bp indels, can abolish miRNA function. Consistent with this, further analysis of multiple *OsMIR528-sgRNA01-15* T1 lines showed that either 4-bp or 54-bp deletions at *OsMIR528*'s mature miRNA region destroyed function (Figure 3C). Presumably, knocking out *OsMIR528* should antagonize repression at some of its target genes. We measured transcription of five putative *OsMIR528* target genes (Yuan et al., 2015; Wu et al., 2017; http://bioinformatics.cau.edu.cn/PMRD/) by quantitative reverse transcription PCR (qRT-PCR) under normal and salt stress conditions. Transcripts of four genes (*Os06g0567900, Os08g0137400, Os06g0154200*, and *Os07g0570550*) were significantly elevated under salt stress in the mutant. For *Os07g0570550*, expression was significantly higher in the mutant than in WT with or without salt stress (Figure 3D). Additionally, we analyzed a putative target gene of *OsMIR408, Os03g0259100*. Using RT-PCR, we detected expression only in the *OsMIR408-03-19* mutant, but not in WT (Supplementary Figure 12). This data supports *Os03g0259100* as a target gene of *OsMIR408*. Taken together, we demonstrated CRISPR-Cas9 as a useful reverse genetic tool for studying function of miRNAs and validating their target genes in rice.

### Single-bp indels lead to the production of functionally redundant new miRNAs

**Figure 4:**
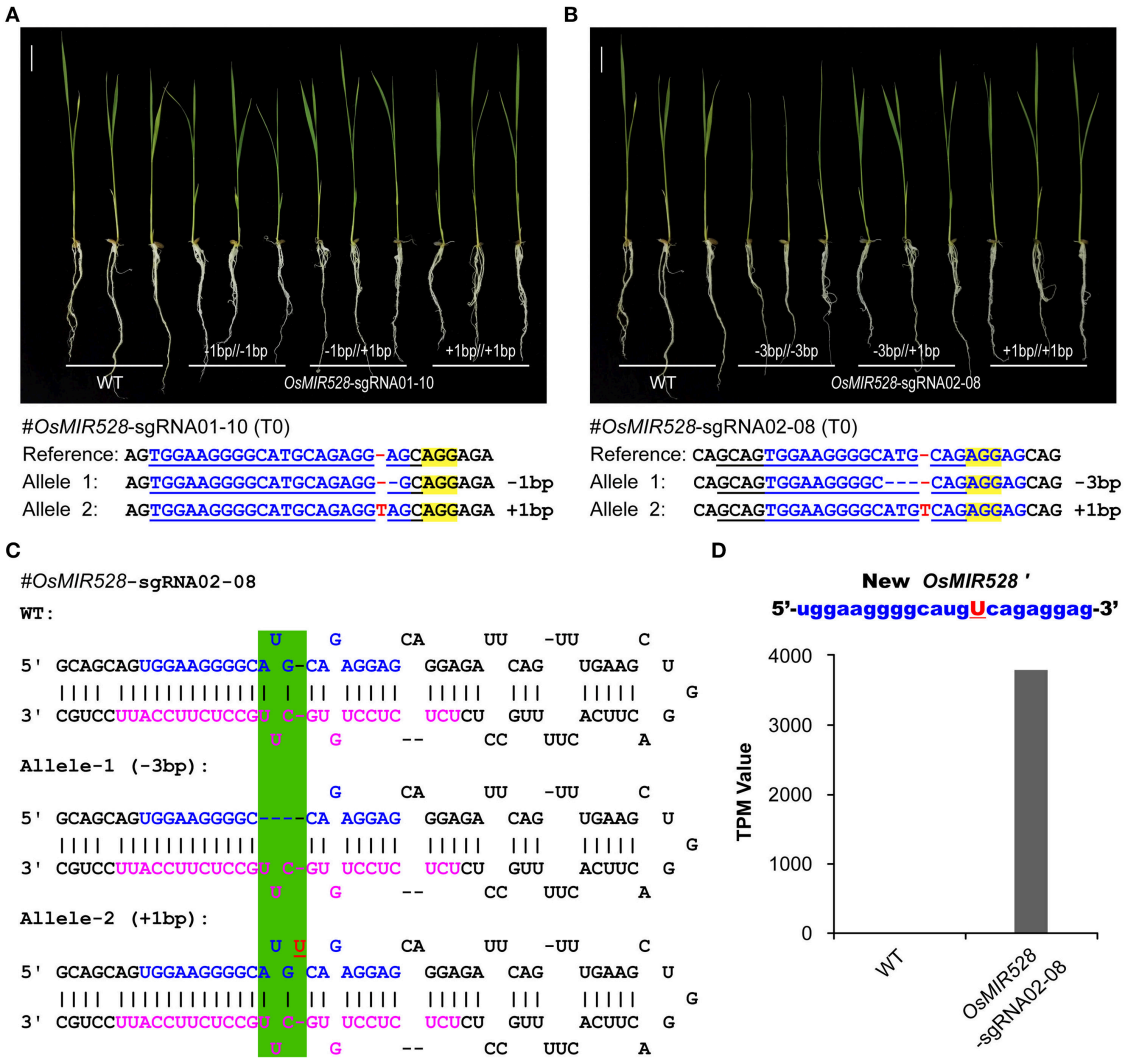
Generation of functionally redundant new microRNAs with genome editing. **(A)** 1-bp indels in mature *OsMIR528* don't abolish miRNA function. **(B)** 3-bp deletion in mature *OsMIR528* abolishes function. **(C)** Illustration of pri-MIR528 produced in *OsMIR528-sgRNA02-08* mutant lines. Mutagenized target regions are highlighted in green shade. **(D)** Detection of a new microRNA, *OsMIR528*′, in the 1-bp insertion mutant line by RNA-seq. TPM, transcripts per million.

*OsMIR528-sgRNA01-10* mutant lines showed the same phenotype to the WT upon salt stress (Figures 3A,B) and further qRT-PCR analysis revealed no significant difference at multiple OsMIR528 target genes across mutant lines and WT (Supplementary Figure 13A), suggesting 1-bp indels in the mature *miRNA* region didn't impact function. We further genotyped many T1 individuals of this line and found all three mutant types (−1/−1 bp; −1/+1 bp; +1/+1 bp) showed WT-like response under salt stress (Figure 4A). To further explore 1-bp indel impact on *OsMIR528* function, we examined T1 lines of *OsMIR528-sgRNA02-08*, which segregated two small indels (−3 and +1 bp) produced by a different sgRNA. The analysis showed that 3-bp deletion, not the 1-bp insertion, destroyed *OsMIR528* function in salt stress response (Figure 4B) and its regulation on target genes (Supplementary Figure 13B). We reasoned that a deletion of 3 bp could be large enough to impact formation of a proper pri-miRNA structure and subsequently the production of correct miRNAs (Figure 4C). On the contrary, 1-bp indels may not impact the process of miRNA maturation (Figure 4C) and even miRNA function due to tolerance of mismatches between miRNA and cognate target genes. To test this hypothesis, we conducted RNA sequencing (RNA-seq) of small RNAs in the biallelic background (−3/+1 bp) of *OsMIR528-sgRNA02-08*. Indeed, we could not detect the potentially new miRNA derived from the 3-bp deletion locus. However, we recovered a new miRNA (named as miRNA528′) of 22 nt, which contained an additional “U” due to the 1-bp insertion (Figure 4D). Presumably, this new *miRNA528* plays an equal or similar role to that of *miRNA528* in regulating genes involved in salt stress response in rice.

### Knocking out a single miRNA leads to expression perturbation of other miRNAs

**Figure 5:**
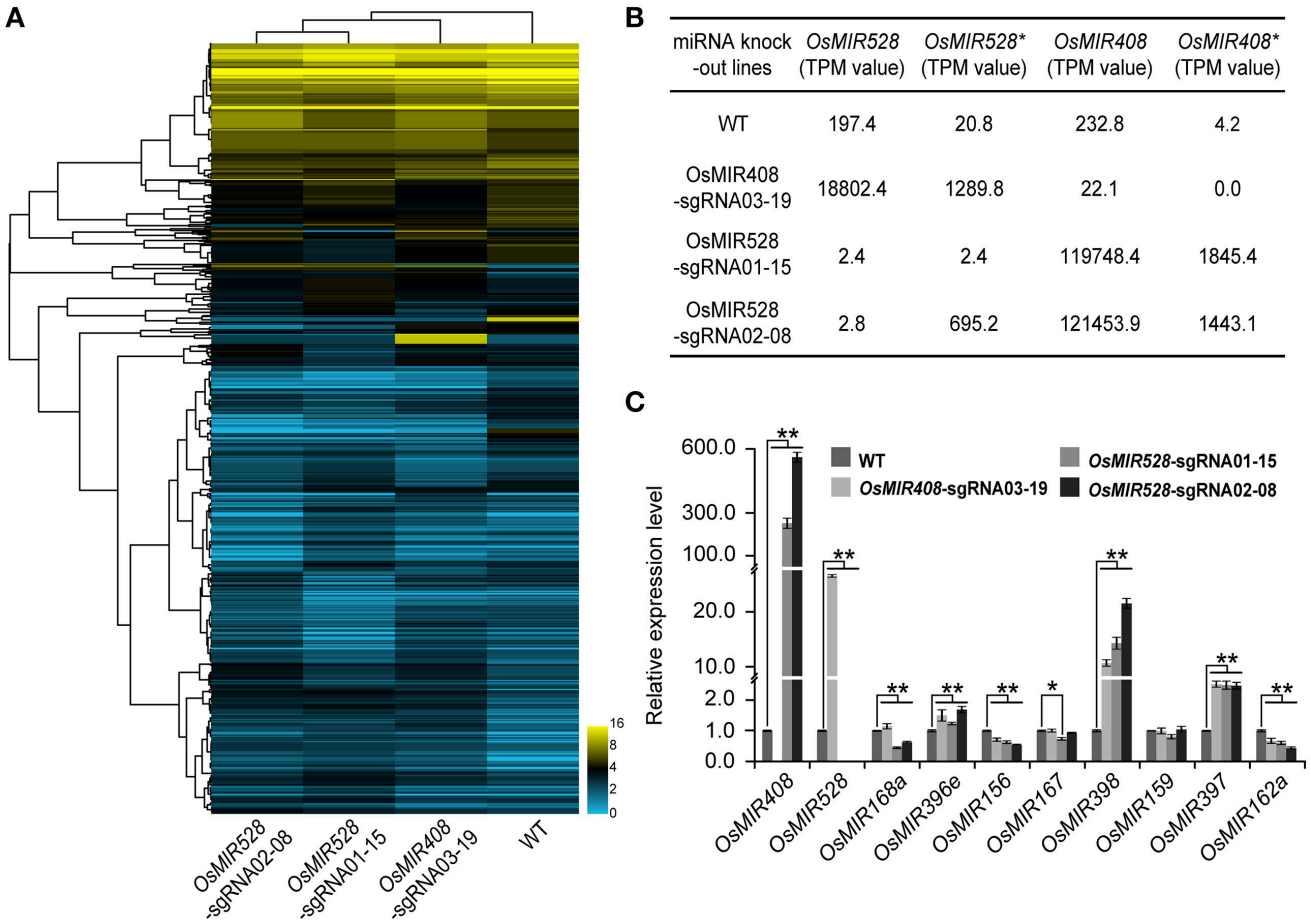
Targeted knocking out specific microRNAs results in global expression perturbation of microRNAs and their putative targets. **(A)** Targeted knock out of *OsMIR408* and *OsMIR528* results in global expression and perturbation of microRNAs. **(B)** *OsMIR408* and *OsMIR528* induce each other as revealed by RNA-seq analysis. **(C)** Knocking out *OsMIR408* or *OsMIR528* boosts expression of *OsMIR397* and *OsMIR398* as validated by qRT-PCR analysis. Asterisks indicate significant differences according to Student's *t*-test; ^*^P ≤ 0.05; ^**^P ≤ 0.01.

miRNAs play critical roles in regulating expression of many peripheral genes involved in different biological processes. Transcription profiling in a miRNA knockout background should reveal regulatory functions of miRNAs. To explore this prospect, we extended our miRNA-seq experiments for detection and quantification of small RNAs in WT and biallelic T1 mutants of *OsMIR408-sgRNA03-19* (−13/−8 bp), *OsMIR528-sgRNA01-15* (−54/−4 bp), and *OsMIR528-sgRNA-02-08* (−3/+1 bp) (Figure 5A). In WT plants, 197.4 transcripts per million (TPM) of *OsMIR528* and 232.8 TMP of *OsMIR408* were detected, while their corresponding miRNA^*^ transcripts were of low abundance: 20.8 TPM and 4.2 TMP, respectively. In *OsMIR408-sgRNA03-19* lines, we couldn't detect any MIR408^*^ transcripts because large deletions (−13 or −8 bp) had destroyed the OsMIR408^*^ region. The large deletion in OsMIR408^*^ appeared to greatly impact the production of mature *OsMIR408* as miRNA abundance was reduced over 10-fold in the mutants to 22.1 TPM. Interestingly, we found expression of OsMIR528 and OsMIR528^*^ were drastically increased (95-fold and 63-fold, respectively) in the *MIR408* knockout background. It was also true of reciprocal experiments where expression of OsMIR408 and OsMIR408^*^ were greatly increased (514-fold and 439-fold, respectively) in *OsMIR528-sgRNA01-15* (−54/−4 bp) mutants, in which transcript of OsMIR528 and OsMIR528^*^ were almost undetectable. In the mutant, *OsMIR528-sgRNA02-08* (−3/+1 bp), in which we discovered a novel miRNA (Figure 4D), expression of OsMIR408 and OsMIR408^*^ also showed significant increases of 522-fold and 344-fold, respectively. As expected, OsMIR528 was almost undetectable in this mutant. However, expression of OsMIR528^*^ is 30 times higher in the mutant than in WT, consistent with the production of new OsMIR528 at a much higher titer than in this mutant background (Figures 4D,5A).

The interesting observation regarding induction of *OsMIR408* in *OsMIR528* mutants and vice versa triggered us to further look at cross regulation among miRNAs at the genome level. Using our miRNA-seq data set for small RNAs, we identified 2,280 putative miRNAs across all samples. We confirmed 551 out of 592 previously validated miRNAs in rice that were available at miRbase (Griffiths-Jones et al., 2008), suggesting a high coverage in our study. We then compared the expression of all identified miRNAs in the WT and mutant backgrounds. Using a stringent criterion of 3-fold cutoff threshold, we identified a list of differentially expressed miRNAs in the *OsMIR408* and *OsMIR520* mutant backgrounds (Supplementary Table 5). In *OsMIR408-sgRNA03-19* mutant, 9 miRNAs were upregulated and 8 miRNAs were downregulated. In *OsMIR528-sgRNA01-15* mutant, 8 miRNAs were upregulated and 26 miRNAs were downregulated. In *OsMIR528-sgRNA02-08* mutant, 11 miRNAs were upregulated and 23 miRNAs were downregulated. Interestingly, the differentially expressed miRNAs were always induced in the same direction, either up or down, in the mutants of *OsMIR408* and *OsMIR528* (Supplementary Table 5). To further validate the data, we conducted qRT-PCR analysis of four upregulated miRNAs (*OsMIR408, OsMIR528, OsMIR397*, and *OsMIR398*), along with 6 control miRNAs. The qRT-PCR data confirmed the results from the RNA-seq analysis (Figures 5B,C).

### Effective deletion of miRNA loci by two DNA DSBs

Our data on *OsMIR528* suggest larger deletions are an effective means for achieving miRNA knockout. However, small indels in either the mature miRNA or miRNA^*^ region could have an unpredictable effect on miRNA function. We reasoned that the best way to knock out a miRNA gene is to completely delete the locus by creating large chromosomal deletions, which requires introduction of two DNA DSBs simultaneously. We constructed two T-DNA vectors for generating large deletions at *OsMIR408* and *OsMIR528* loci. Rice protoplasts transformed with these constructs were used for DNA extraction and PCR, and PCR products were then cloned into TA cloning vectors. By screening many individual clones using PCR, smaller products representing large chromosomal deletion events were readily discovered (Supplementary Figures 14A,B). These deletions along with other mutations were further confirmed by Sanger sequencing (Supplementary Figures 14A,B). In both cases, around 20% of tested clones carried large deletions (Supplementary Figure 14C).

We next examined large deletion frequencies at both loci in stable transgenic rice plants. Among 20 T0 lines for the *OsMIR408* locus, 12 lines contained large deletions and 1 line was homozygous. Interestingly, a single line (#OsMIR408-sgRNA02+sgRNA04-11) contained one large deletion and one inversion (Table 2, Supplementary Figures 15A, 16). Among 22 T0 lines for the *OsMIR528* locus, 5 lines contained large deletions with two of them being homozygous and one line (#OsMIR528-sgRNA01+sgRNA03-07) contained an inversion (Table 2, Supplementary Figures 15B, 17). These results suggest *miRNA* loci can be effectively deleted by introducing two DNA DSBs with multiplexed CRISPR-Cas9.

**Table 2:** Summary of mutation frequencies at targeted miRNA loci in T0 lines that express two sgRNAs.

**Targeted rice pri-microRNA locus**	**Transgenetic CRISPR/Cas9 vector**	**Tested T0 seedling**	**Deletion T0 seedling mutant: number, ratio**	**Double NHEJ T0 seedling mutant: number, ratio**	**Single NHEJ T0 seedling mutant: number, ratio**
*OsMIR408*	pZmUbi1::Cas9+OsU6::*OsMIR408*−sgRNA02+sgRNA04 (pZJP025)	20	12, 60.0%	8, 40.0%	0, 0.0%
*OsMIR528*	pZmUbi1::Cas9+OsU6::*OsMIR528*−sgRNA01+sgRNA03 (pZJP026)	22	5, 22.7%	13, 69.1%	1, 4.6%

## Discussion

Unlike other protein-coding genes, mature miRNAs are only about 20- to 24-nucleotide in length (Bartel, 2004; Voinnet, 2009). We have checked the PAM (NGG) of all 592 rice miRNAs at miRBase (http://www.mirbase.org/) and found that 556 miRNAs (93.92%) harbored suitable PAM sites. Only 36 miRNAs (6.08%) did not contain suitable PAM sequences. In this report, we demonstrated that mature miRNA or miRNA^*^ can be targeted using CRISPR-Cas9 with a single sgRNA and there is no distinct difference of the efficiency of targeting of miRNA and miRNA^*^(Table 1) (in principle, targeted mutagenesis efficiency for miRNA or miRNA^*^ DNA sequence is determined by the designed sgRNAs, which may vary from case to case). In most cases, we could not use RFLP for detecting mutations at target sites because it is sufficiently rare to find a restriction enzyme cleavage site that overlaps with Cas9 cleavage sites within these narrowly defined regions. Recently, we reported SSCP as an alternative genotyping method for screening CRISPR-Cas9 induced mutations (Zheng et al., 2016). Here, we again further demonstrated that SSCP is well-suited for genotyping miRNA mutants with small indels. Due to high efficiency of CRISPR-Cas9 based genome editing in rice, the advantage of SSCP may not be apparent as Sanger sequencing can be directly applied for genotyping mutant lines. However, we note that genome editing frequency in many other plant species is not as high as in rice (Paul and Qi, 2016). To save time and cost, SSCP could be an important method for rapid screening of many candidate genome editing lines prior to Sanger sequencing in these plant species.

A series of miRNA mutants of *OsMIR408* and *OsMIR528* generated in this work provided a valuable resource for functional study of these two important microRNAs. By linking genotype to phenotype among mutants of *OsMIR528*, we found that 1-bp indels at the mature *miRNA* or miRNA^*^ region failed to abolish miRNA function (Figures 3, 4). Interestingly, new functionally redundant miRNAs could be produced in such mutants. This is consistent with the fact that miRNAs can tolerant mismatches within target mRNA in plants (Wang et al., 2015). Conceivably, HDR based precise genome editing of endogenous miRNA loci should represent a new strategy for producing artificial miRNAs with new targeting specificity and function.

Knocking out miRNAs provides opportunities to validate their target genes. A major role of miRNAs in plants is to down-regulate target mRNAs. By genetically disrupting a miRNA, transcripts of cognate target genes may be upregulated. Following this principle, we validated three potential target genes of *OsMIR528* and one potential target gene of *OsMIR408*. While we were preparing this manuscript, it was reported that *OsMIR528* negatively regulates virus defense through repressing the target gene encoding an L-ascorbate oxidase (AO) (Wu et al., 2017). In a T-DNA knockout line of *OsMIR528*, reactive oxygen species (ROS) was elevated, leading to enhance resistance to viruses (Wu et al., 2017). Consistently, we also validated this *AO* gene (*Os06g0567900*) as one of *OsMIR528*'s targets (Figure 3D). Previously, it was shown that overexpression of *OsMIR528* in Creeping Bentgrass resulted in reduced AO expression and increased ROS detoxification (Yuan et al., 2015). Taken together, these studies support that *OsMIR528* plays an important role in plant biotic and abiotic stress responses by controlling ROS. In the future, it will be interesting to study additional target genes of *OsMIR528* (Figure 3D) and *OsMIR408* (Supplementary Figure 12), which should help elucidate biological functions of these important miRNAs.

Another interesting finding in this study shows that knocking out either *OsMIR408* and *OsMIR528* results in expression profile changes of multiple other miRNAs. Of note, these miRNAs with altered expression were either upregulated or downregulated in the same direction in mutants of *OsMIR408* and *OsMIR528*. Although both miR408 and miR528 were proposed to function in stress responses in plants (Cheah et al., 2015; Ma C. et al., 2015; Sharma et al., 2015), there has been no firm evidence supporting they function in the same pathway. Further, although *OsMIR528-sgRNA0208* (−3/+1 bp) is phenotypically similar to WT upon salt stress, expression of many miRNAs was significantly altered in this background. It thus appears that biogenesis of many miRNAs in plants is interrelated. This is a highly significant finding as it may complicate our analysis of miRNAs in genetic knockout lines. Hence, it will be important to further investigate the molecular basis of such cross-regulation among miRNAs in rice and possibly other plants. Interestingly, a very recent study in Arabidopsis revealed that loss of miR159 increases miR156 level and the repression of miR156 by miR159 is largely mediated by MYB33, an R2R3 MYB domain transcription factor targeted by miR159 (Guo et al., 2017). It will be interesting to see whether cross-regulation among miRNAs in rice is based on a similar mechanism. It will be very useful to draw miRNA-miRNA regulation networks (Akdogan et al., 2016) toward understanding the underlining mechanisms.

Finally, we demonstrate that a miRNA family of multiple members could be effectively and simultaneously mutagenized by CRISPR-Cas9. By targeting *OsMIR815* and *OsMIR820* families, each containing three members, biallelic triple mutants were readily obtained in the T0 generation. Recently, miRNA gene families have been targeted using multiple sgRNAs simultaneously in zebrafish (Narayanan et al., 2016). With multiplexed CRISPR-Cas9 systems like the one we recently developed (Lowder et al., 2015), it should be feasible to simultaneously knock out multiple miRNA families for genetic analysis in plants.

## Conclusion

Our work demonstrated promising applications of CRISPR-Cas9 for editing miRNA genes and gene families for basic and applied research in rice and other plants.

## Author contributions

YZ, JZ, and YQ designed the experiments. JZ, YZ, XT, and KD generated all Vectors. ZZ, XT, XZ, and KD performed the transient assays in protoplasts and prepared samples for small RNA sequencing. YZ, JZ, TZ, and YQ analyzed the deep sequencing data. JZ, KD, YC, ZZ, and LT generated stable transgenic rice and analyzed the plants. JZ performed the RT-PCR and real-time PCR. AT produced the chlorophyll content analysis. YQ, YZ, JZ, and KD wrote the paper with input from other authors. All authors read and approved the final manuscript.

### Conflict of interest statement

The authors declare that the research was conducted in the absence of any commercial or financial relationships that could be construed as a potential conflict of interest.
