# Supplementary material for: CRISPR-Cas9 Based Genome Editing Reveals New Insights into MicroRNA Function and Regulation in Rice
Source: Front Plant Sci. 2017 Sep 13;8:1598. doi: 10.3389/fpls.2017.01598 (PMC5602353; doi:10.3389/fpls.2017.01598)
Supplement: Supplementary file 1 [file Image1.PDF]

Supplementary Fig 1. Eight rice microRNAs targeted by CRISPR-Cas9

```
>OsMIR408
            (OsMIR408-sgRNA02)GGATGAGGCAGAGCATGGGATGG
GGGTCTGTGATTGGAGAGGAGAGGAGACAGGGATGAGGCAGAGCATGGGATGGGGCTATCAACAGATGTAGATTATTCCTTGCACAAGAGATGATGATGAGCTGTGAATGAGTTCTGAGAGTAGATGGCT
GGTGTTGTTGTTGCTCCCTCCCCTGCACTGCCTCTTCCCTGGCTCCCCTGCACACCTCTCTCTCTCTCTCTCTCTGTGT
                   CCCCTGCACTGCCTCTTCCCTGG(OsMIR408-sgRNA03)
               CCCTCCCCTGCACTGCCTCTTCC(OsMIR408-sgRNA04)

5' --GGGAGUUCUGUGAUUGGAGAGGAGAGGAGA      U       A   U    U     UAU      AUGUAGAUUAUUCCUUGCACAAGAGAUGAUGA
                                    CAGGGA GAGGCAG GCA GGGA GGGGC   CAACAG                                 U
                                    |||||| ||||||| ||| |||| |||||   ||||||
                                    GUCCCU CUCCGUC CGU CCCU CCUCG   GUUGUU                                 G
3' UCUCUCUCUCUCUCUCUCCACACGUCCCCUCG      U       A   C    C     -UU      GUGGUCGGUAGAGAGUCUUGAGUAAGUGUCGA
```

```
>OsMIR528
GCAGCAGTGGAAGGGGCATGCAGAGGAGCAGGAGATTCAGTTTGAAGCTGGACTTCACTTTTGCCTCTCTCTCCTGTGCTTGCCTCTTCCATTCCTGC
        GAAGGGGCATGCAGAGGAGCAGG(OsMIR528-sgRNA01)     (OsMIR528-sgRNA03)CCTGTGCTTGCCTCTTCCATTCC
   GCAGTGGAAGGGGCATGCAGAGG(OsMIR528-sgRNA02)

                     U   G     CA     UU   -UU     C
5' GCAGCAGUGGAAGGGGCA GCA AGGAG  GGAGA  CAG   UGAAG  U
   |||| ||||||||||||| ||| |||||  |||||  |||   |||||    G
3' CGUCCUUACCUUCUCCGU CGU UCCUC  UCUCU  GUU   ACUUC  G
                     U   G     --     CC   UUC     A
```

```
>OsMIR815a
TTTTTTTAGACTCTTAATTTCTTTTTCTAGGATGAACTCTTAGGGTGTGTTTGAGGAGAAGGGGATTGAGGAGATTGGGGAGA
                                    (OsMIR815-sgRNA01)GAGAAGGGGATTGAGGAGATTGG

            A   -     UU        -A     A
5' UUUUUUUAG CUC UUAAU  CUUUUUCU  GGAUG ACUCU
   ||||||||| ||| |||||  ||||||||  ||||| ||||| U
3' AGAGGGGUU GAG AGUUA  GGAAGAGG  UUUGU UGGGA
            A   G     GG        AG     G
```

```
>OsMIR815b
CTAATGGTTCACCTCGTTTTGCGTATATTCCCAATCTTCTCTATTTCCTTCTCCTCAAACACAGCCTGGGAGTGTTTGAGAAGGGGATTGAGGAGATTGGGAAGATACGTAAAACGAGGTGAGCCATTAG
                                                                      (OsMIR815-sgRNA01)GAGAAGGGGATTGAGGAGATTGG

                             A              U  UU       CUC      AG C
5' CUAAUGGUUCACCUCGUUUUGCGUAU UUCCCAAUCUUCUC AU  CCUUCUC   AAACAC  C U
   |||||||||||||||||||||||||| |||||||||||||| ||  |||||||   ||||||  |
3' GAUUACCGAGUGGAGCAAAAUGCAUA AAGGGUUAGAGGAG UA  GGAAGAG   UUUGUG  G G
                             G              U  GG       ---      -A G
```

```
>OsMIR815c
CTTCTCCTCTCTTCTCTCCTCCACCTCAGCATTTAGCCGGCTTATAGCCTATACTTCCTCTAAGGGTGTGTTTGAGGAGAAGGGGATTGAGGAGATTGGGAAG
                                                                     (OsMIR815-sgRNA01)GAGAAGGGGATTGAGGAGATTGG

         -C       --      C A      CAUUUAGCCGGCUUAUAGCC
5' CUUCUC  UCUCUUC  UCUCCU C CCUCAG                    U
   ||||||  |||||||  |||||| | ||||||
3' GAAGGG  AGAGGAG  AGGGGA G GGAGUU                    A
         UU       UU      A A      UGUGUGGGAAUCUCCUUCAU
```

```
>OsMIR820a
   GTCGGCCTCGTGGATGGACCAGG (OsMIR820-sgRNA01)
TGCGTCGGCCTCGTGGATGGACCAGGAGCTCAACATTCCTTAAGGTTGTTCTTTCAAACCCATACAAGGTTCCACCGCCTGCATTGTTCCAAGAGTGTCTTGGATGAAAGTAGGAAGTGGAACCTTGTTA
GGGTTGGAACGAACTGCCTTAAGGAAGTCGATGCTCCAGGTCCGTCCAGGAGGACGACTCA

   U C    G    G          A     -  A  A           U    U   A     AU            CG     A  GU       A
5'  G GUCG CCUC UGGAUGGACC GGAGC UC AC UUCCUUAAGGU GUUC UUC AACCC  ACAAGGUUCCAC  CCUGC UU  UCCAAG G
    | |||| |||| |||||||||| ||||| || || ||||||||||| |||| ||| |||||  ||||||||||||  ||||| ||  |||||| U
3'  C CAGC GGAG ACCUGCCUGG CCUCG AG UG AAGGAAUUCCG CAAG AAG UUGGG  UGUUCCAAGGUG  GGAUG AA  AGGUUC G
   A U    A    G          A     U  C  -           U    C   G     AU            AA     A  GU       U
```

```
>OsMIR820b
        GTCGGCCTCGTGGATGGACCAGG (OsMIR820-sgRNA01)
GTACATGCGTCGGCCTCGTGGATGGACCAGGAGCTCGACGTTCCTTAAGGCCGTTCTTTCCGACCCATACAAGGTTCCGTCGCCTGCATTGTTCCAAGAGTGTCTTGGACGAAAGCAGGAAGTGGAACCT
TGTTAAGGTCGGAACGAACTGCCTTAAGGAAGTCGATGCTTTAGGTCCGTCCACGAGGACGACGCAGCTAG

   GUACA       G               AG    -     G           C    U        CAU            CG     -A     U     A
5'      UGCGUCG CCUCGUGGAUGGACC  GAGC UCGAC UUCCUUAAGGC GUUC UUCCGACC   ACAAGGUUCCGU  CCUGC  UGUG CCAAG G
        ||||||| |||||||||||||||  |||| ||||| ||||||||||| |||| ||||||||   ||||||||||||  |||||  |||| ||||| U
3'      ACGCAGC GGAGCACCUGCCUGG  UUCG AGCUG AAGGAAUUCCG CAAG AAGGCUGG   UGUUCCAAGGUG  GGACG  AGCA GGUUC G
   GAUCG       A               AU    U     -           U    C        AAU            AA     AA     -     U
```

```
>OsMIR820c
   GTCGGCCTCGTGGATGGACCAGG (OsMIR820-sgRNA01)
TGCGTCGGCCTCGTGGATGGACCAGGAGCTCGACATTCCTTAAGGTCGTTCTTTCCGACCCATACAAGGTTCCGCTGCCTGCATTGTTCCAAGAGTGTCTTGGACAAAAGCAGGAAGTGGAACCTTGTTA
AGGTCGGAACGAACTGCCTTAAGGAAGTCGATGCTCCAGGTCCGTCCACGAGGACGACGCA

   U      G               A     -     A           C    U        CAU             G     -A    U      A
5'  GCGUCG CCUCGUGGAUGGACC GGAGC UCGAC UUCCUUAAGGU GUUC UUCCGACC   ACAAGGUUCCGCU CCUGC  UUGU CCAAG G
    |||||| ||||||||||||||| ||||| ||||| ||||||||||| |||| ||||||||   ||||||||||||| |||||  |||| ||||| U
3'  CGCAGC GGAGCACCUGCCUGG CCUCG AGCUG AAGGAAUUCCG CAAG AAGGCUGG   UGUUCCAAGGUGA GGACG  AACA GGUUC G
   A      A               A     U     -           U    C        AAU             A     AA    -      U
```

Supplementary Fig 2. CRISPR-Cas9 induced mutagenesis at target miRNA loci in transformed rice protoplasts

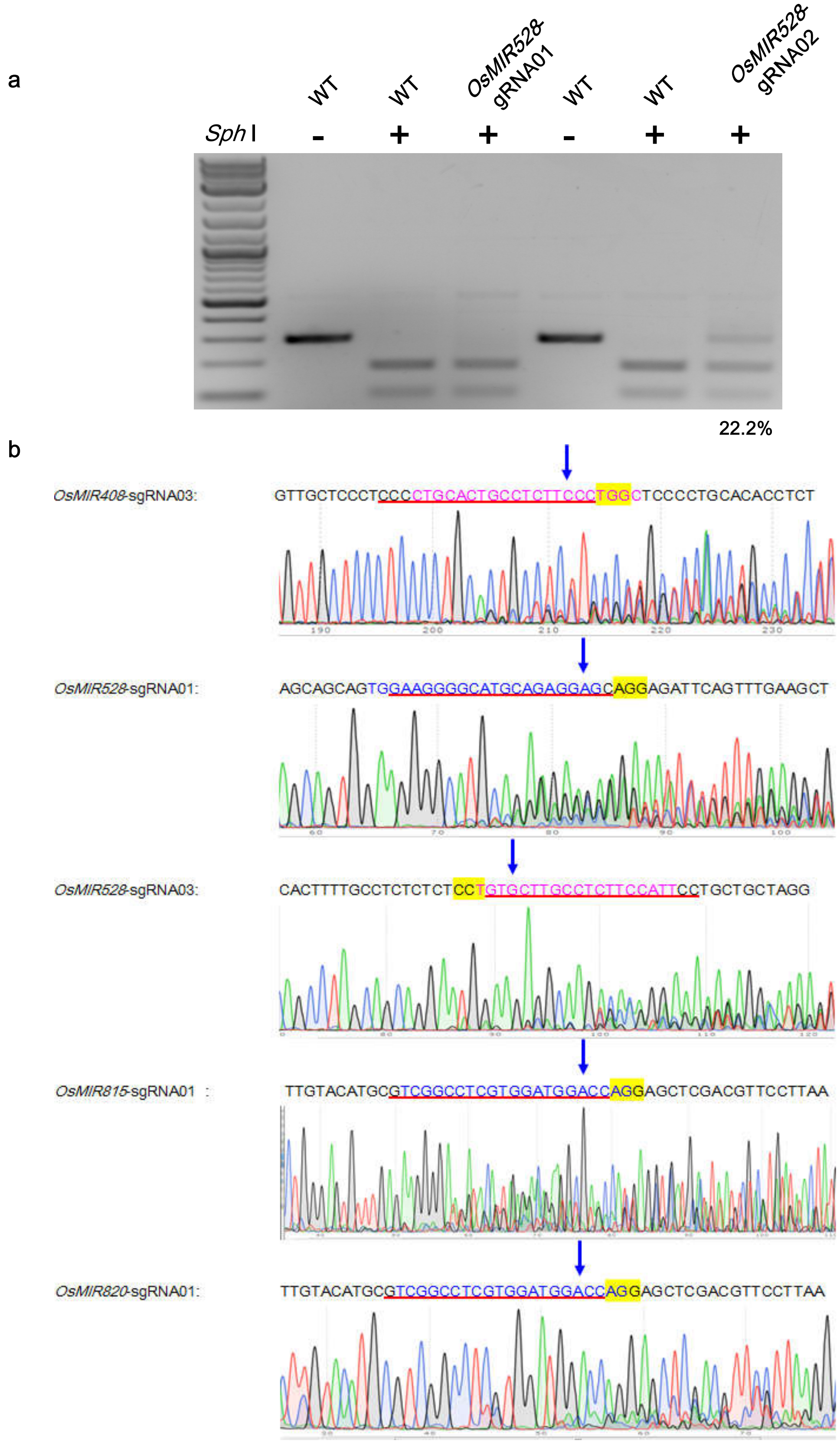

Supplementary Fig 3. Screen for genome edited T0 lines targeted with *OsMIR528*-sgRNA01 by SSCP

a

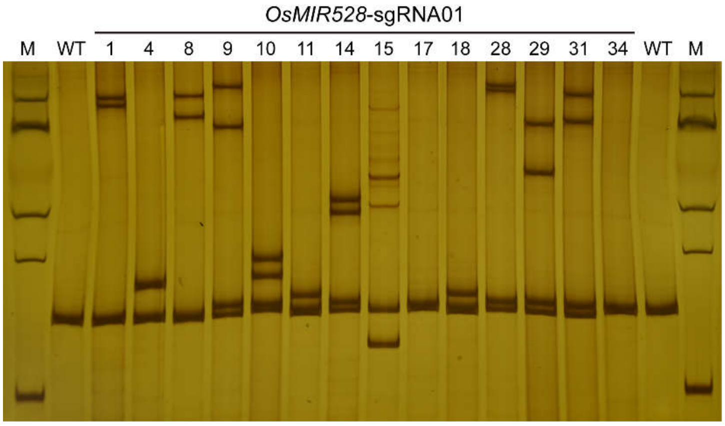

b

```
Reference:      AGCAGCAGTGGAAGGGGCATGCAGAGG-AGCAGGAGATTCAGTTTGAAGCT
#OsMIR528-sgRNA01-01
Allele 1:       AGCAGCAGTGGAAGGGGCA---------AGCAGGAGATTCAGTTTGAAGCT (-8  bp)
Allele 2:       AGCAGCAGTGGAAGGGGCATGCAGA--TAGCAGGAGATTCAGTTTGAAGCT (-2/+1  bp)
#OsMIR528-sgRNA01-04
Allele 1:       AGCAGCAGTGGAAGGGGCATGCAGAG--AGCAGGAGATTCAGTTTGAAGCT (-1  bp)
Allele 2:       AGCAGCAGTGGAAGGGGCATGCAGAG----CAGGAGATTCAGTTTGAAGCT (-3  bp)
#OsMIR528-sgRNA01-08
Allele 1:       AGCAGCAGTGGAAGGGGCATGCAG----AGCAGGAGATTCAGTTTGAAGCT (-3  bp)
Allele 2:       AGCAGCAGTGGAAGGGGCATGCAG------------------TTTGAAGCT (-17 bp)
#OsMIR528-sgRNA01-09
Allele 1:       AGCAGCAGTGGAAGGGGCATGCAG-----GCAGGAGATTCAGTTTGAAGCT (-4  bp)
Allele 2:       AGCAGCAGTGGAAGGGGCATGCAGAGGGAGCAGGAGATTCAGTTTGAAGCT (+1  bp)
#OsMIR528-sgRNA01-10
Allele 1:       AGCAGCAGTGGAAGGGGCATTCAGAGG--GCAGGAGATTCAGTTTGAAGCT (-1  bp)
Allele 2:       AGCAGCAGTGGAAGGGGCATGCAGAGGTAGCAGGAGATTCAGTTTGAAGCT (+1  bp)
#OsMIR528-sgRNA01-11
Allele 1:       AGCAGCAGTGGAAGGGGCATGCAGA---AGCAGGAGATTCAGTTTGAAGCT (-2  bp)
Allele 2:       AGCAGCAGTGGAAGGGGCATGCAGA----GCAGGAGATTCAGTTTGAAGCT (-3  bp)
#OsMIR528-sgRNA01-14
Allele 1:       AGCAGCAGTGGAAGGGGCATGCAGAG---GCAGGAGATTCAGTTTGAAGCT (-2  bp)
Allele 2:       AGCAGCAGTGGAAGGGGCATGCAGAGGTAGCAGGAGATTCAGTTTGAAGCT (+1  bp)
#OsMIR528-sgRNA01-15
Allele 1:       -------------------------------------------TTGAAGCT (-54 bp)
Allele 2:       AGCAGCAGTGGAAGGGGCATGCA-----AGCAGGAGATTCAGTTTGAAGCT (-4  bp)
#OsMIR528-sgRNA01-17
Allele 1:       AGCAGCAGTGGAAGGGGCATGCAGAG--AGCAGGAGATTCAGTTTGAAGCT (-1  bp)
Allele 2:       AGCAGCAGTGGAAGGGGCATGCAGAG--AGCAGGAGATTCAGTTTGAAGCT (-1  bp)
#OsMIR528-sgRNA01-18
Allele 1:       AGCAGCAGTGGAAGGGGCATGCAGAGG--GCAGGAGATTCAGTTTGAAGCT (-1  bp)
Allele 2:       AGCAGCAGTGGAAGGGGCATGCAG--G-AGCAGGAGATTCAGTTTGAAGCT (-2  bp)
#OsMIR528-sgRNA01-28
Allele 1:       AGCAGCAGTGGAAGGGGCATGCAG------CAGGAGATTCAGTTTGAAGCT (-5  bp)
Allele 2:       AGCAGCAGTGGAAGGGGCATGCAGAGGAGTCAGGAGATTCAGTTTGAAGCT (+1  bp)
#OsMIR528-sgRNA01-29
Allele 1:       AGCAGCAGTGGAAGGGGCATGCAGAG----CAGGAGATTCAGTTTGAAGCT (-3  bp)
Allele 2:       AGCAGCAGTGGAAGGGGCATGCAGAGGAGTCAGGAGATTCAGTTTGAAGCT (+1  bp)
#OsMIR528-sgRNA01-31
Allele 1:       AGCAGCAGTGGAAGGGGCATGCA-----AGCAGGAGATTCAGTTTGAAGCT (-4  bp)
Allele 2:       AGCAGCAGTGGAAGGGGCATGCAGAGGCAGCAGGAGATTCAGTTTGAAGCT (+1  bp)
```

Supplementary Fig 4. Screen for genome-edited T0 lines T0 targeted with *OsMIR528*-sgRNA03 by SSCP

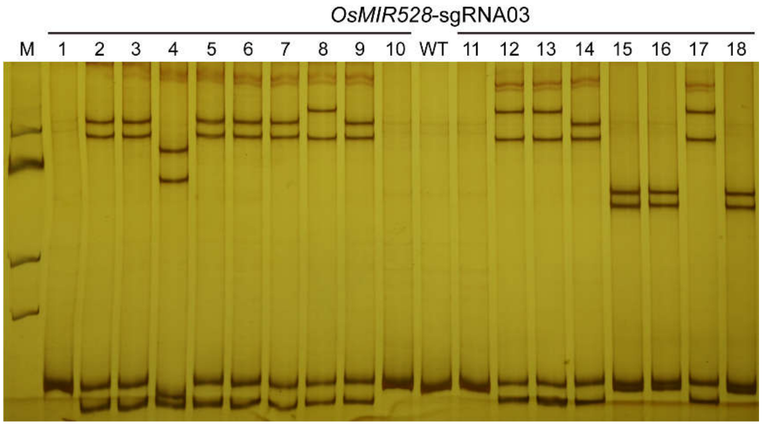

Supplementary Fig 5. Sanger sequencing of genome edited T0 lines targeted with *OsMIR528*-sgRNA03

```
Reference:    GCCTCTCTCTCCTGTG-CTTGCCTCTTCCATTCCTGCTGCTAGGCTGTTCT
#OsMIR528-sgRNA03-01
Allele 1:     GCCTCTCTCTCCTGTGCCTTGCCTCTTCCATTCCTGCTGCTAGGCTGTTCT (+1  bp)
Allele 2:     GCCTCTCTCTCCTGTGTCTTGCCTCTTCCATTCCTGCTGCTAGGCTGTTCT (+1  bp)
#OsMIR528-sgRNA03-02
Allele 1:     GCCTCTCTCTCCTG---------------------CTGCTAGGCTGTTCT (-21 bp)
Allele 2:     GCCTCTCTCTCCTGTGTCTTGCCTCTTCCATTCCTGCTGCTAGGCTGTTCT (+1  bp)
#OsMIR528-sgRNA03-03
Allele 1:     GCCTCTCTCTCCTG---------------------CTGCTAGGCTGTTCT (-21 bp)
Allele 2:     GCCTCTCTCTCCTGTGTCTTGCCTCTTCCATTCCTGCTGCTAGGCTGTTCT (+1  bp)
#OsMIR528-sgRNA03-04
Allele 1:     GCCTCTCTCTCCTG---------------------CTGCTAGGCTGTTCT (-21 bp)
Allele 2:     GCCTCTCTCTCCTGTG----GCCTCTTCCATTCCTGCTGCTAGGCTGTTCT (-3  bp)
#OsMIR528-sgRNA03-05
Allele 1:     GCCTCTCTCTCCTG---------------------CTGCTAGGCTGTTCT (-21 bp)
Allele 2:     GCCTCTCTCTCCTGTGTCTTGCCTCTTCCATTCCTGCTGCTAGGCTGTTCT (+1  bp)
#OsMIR528-sgRNA03-06
Allele 1:     GCCTCTCTCTCCTG---------------------CTGCTAGGCTGTTCT (-21 bp)
Allele 2:     GCCTCTCTCTCCTGTGTCTTGCCTCTTCCATTCCTGCTGCTAGGCTGTTCT (+1  bp)
#OsMIR528-sgRNA03-07
Allele 1:     GCCTCTCTCTCCTG---------------------CTGCTAGGCTGTTCT (-21 bp)
Allele 2:     GCCTCTCTCTCCTGTGTCTTGCCTCTTCCATTCCTGCTGCTAGGCTGTTCT (+1  bp)
#OsMIR528-sgRNA03-08
Allele 1:     GCCTCTCTCTCCTG---------------------CTGCTAGGCTGTTCT (-21 bp)
Allele 2:     GCCTCTCTCTCCTGTGACTTGCCTCTTCCATTCCTGCTGCTAGGCTGTTCT (+1  bp)
#OsMIR528-sgRNA03-09
Allele 1:     GCCTCTCTCTCCTG---------------------CTGCTAGGCTGTTCT (-21 bp)
Allele 2:     GCCTCTCTCTCCTGTGTCTTGCCTCTTCCATTCCTGCTGCTAGGCTGTTCT (+1  bp)
#OsMIR528-sgRNA03-12
Allele 1:     GCCTCTCTCTCCTG---------------------CTGCTAGGCTGTTCT (-21 bp)
Allele 2:     GCCTCTCTCTCCTGTGACTTGCCTCTTCCATTCCTGCTGCTAGGCTGTTCT (+1  bp)
#OsMIR528-sgRNA03-13
Allele 1:     GCCTCTCTCTCCTG---------------------CTGCTAGGCTGTTCT (-21 bp)
Allele 2:     GCCTCTCTCTCCTGTGACTTGCCTCTTCCATTCCTGCTGCTAGGCTGTTCT (+1  bp)
#OsMIR528-sgRNA03-14
Allele 1:     GCCTCTCTCTCCTG---------------------CTGCTAGGCTGTTCT (-21 bp)
Allele 2:     GCCTCTCTCTCCTGTGACTTGCCTCTTCCATTCCTGCTGCTAGGCTGTTCT (+1  bp)
#OsMIR528-sgRNA03-15
Allele 1:     GCCTCTCTCTCCTG--A-------CTTCCATTCCTGCTGCTAGGCTGTTCT (-8/+1 bp)
Allele 2:     GCCTCTCTCTCCTGTGACTTGCCTCTTCCATTCCTGCTGCTAGGCTGTTCT (+1  bp)
#OsMIR528-sgRNA03-16
Allele 1:     GCCTCTCTCTCCTG--A-------CTTCCATTCCTGCTGCTAGGCTGTTCT (-8/+1 bp)
Allele 2:     GCCTCTCTCTCCTGTGACTTGCCTCTTCCATTCCTGCTGCTAGGCTGTTCT (+1  bp)
#OsMIR528-sgRNA03-17
Allele 1:     GCCTCTCTCTCCTG---------------------CTGCTAGGCTGTTCT (-21 bp)
Allele 2:     GCCTCTCTCTCCTGTGACTTGCCTCTTCCATTCCTGCTGCTAGGCTGTTCT (+1  bp)
#OsMIR528-sgRNA03-18
Allele 1:     GCCTCTCTCTCCTG--A-------CTTCCATTCCTGCTGCTAGGCTGTTCT (-8/+1 bp)
Allele 2:     GCCTCTCTCTCCTGTGACTTGCCTCTTCCATTCCTGCTGCTAGGCTGTTCT (+1  bp)
```

Supplementary Fig 6. Screen for genome edited lines at the *OsMIR408* Allele by SSCP and Sanger sequencing

a

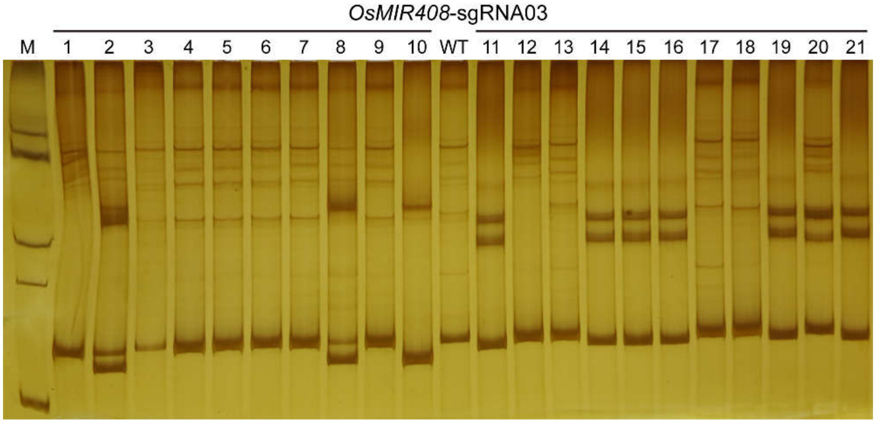

b

```
Reference:     GTTGCTCCCTCCCCTGCACTGCCTCTT-CCCTGGCTCCCCTGCACACCTCT
#OsMIR408-gRNA03-02
Allele 1:      GTTGCTCCCTCCCCTGCAC-------------------------ACCTCT  (-25 bp)
Allele 2:      GTTGCTCCCTCCCCTGCACTGCCTC---CCCTGGCTCCCCTGCACACCTCT (-2  bp)
#OsMIR408-gRNA03-08
Allele 1:      GTTGCTCCCTCCCCTGCAC-------------------------ACCTCT  (-25 bp)
Allele 2:      GTTGCTCCCTCCCCTGCACTGCCTCTT-CCCTGGCTCCCCTGCACACCTCT (WT)
#OsMIR408-gRNA03-10
Allele 1:      GTTGCTCCCTCCCCTGCAC-------------------------ACCTCT  (-25 bp)
Allele 2:      GTTGCTCCCTCCCCTGCACTGCCTCTT-CCCTGGCTCCCCTGCACACCTCT (WT)
#OsMIR408-gRNA03-11
Allele 1:      GTTGCTCCCTCCCC--------------CCCTGGCTCCCCTGCACACCTCT (-13 bp)
Allele 2:      GTTGCTCCCTCCCCTGCAC---------CCCTGGCTCCCCTGCACACCTCT (-8  bp)
#OsMIR408-gRNA03-14
Allele 1:      GTTGCTCCCTCCCC--------------CCCTGGCTCCCCTGCACACCTCT (-13 bp)
Allele 2:      GTTGCTCCCTCCCCTGCAC---------CCCTGGCTCCCCTGCACACCTCT (-8  bp)
#OsMIR408-gRNA03-15
Allele 1:      GTTGCTCCCTCCCC--------------CCCTGGCTCCCCTGCACACCTCT (-13 bp)
Allele 2:      GTTGCTCCCTCCCCTGCAC---------CCCTGGCTCCCCTGCACACCTCT (-8  bp)
#OsMIR408-gRNA03-16
Allele 1:      GTTGCTCCCTCCCC--------------CCCTGGCTCCCCTGCACACCTCT (-13 bp)
Allele 2:      GTTGCTCCCTCCCCTGCAC---------CCCTGGCTCCCCTGCACACCTCT (-8  bp)
#OsMIR408-gRNA03-19
Allele 1:      GTTGCTCCCTCCCC--------------CCCTGGCTCCCCTGCACACCTCT (-13 bp)
Allele 2:      GTTGCTCCCTCCCCTGCAC---------CCCTGGCTCCCCTGCACACCTCT (-8  bp)
#OsMIR408-gRNA03-20
Allele 1:      GTTGCTCCCTCCCCTGCACTGCCTC-----CTGGCTCCCCTGCACACCTCT (-4  bp)
Allele 2:      GTTGCTCCCTCCCCTGCACTGCCTCTTACCCTGGCTCCCCTGCACACCTCT (+1  bp)
#OsMIR408-gRNA03-21
Allele 1:      GTTGCTCCCTCCCC--------------CCCTGGCTCCCCTGCACACCTCT (-13 bp)
Allele 2:      GTTGCTCCCTCCCCTGCAC---------CCCTGGCTCCCCTGCACACCTCT (-8  bp)
```

Supplementary Fig 7. Screen for genome-edited T0 lines targeted with *OsMIR815*-sgRNA01 by SSCP

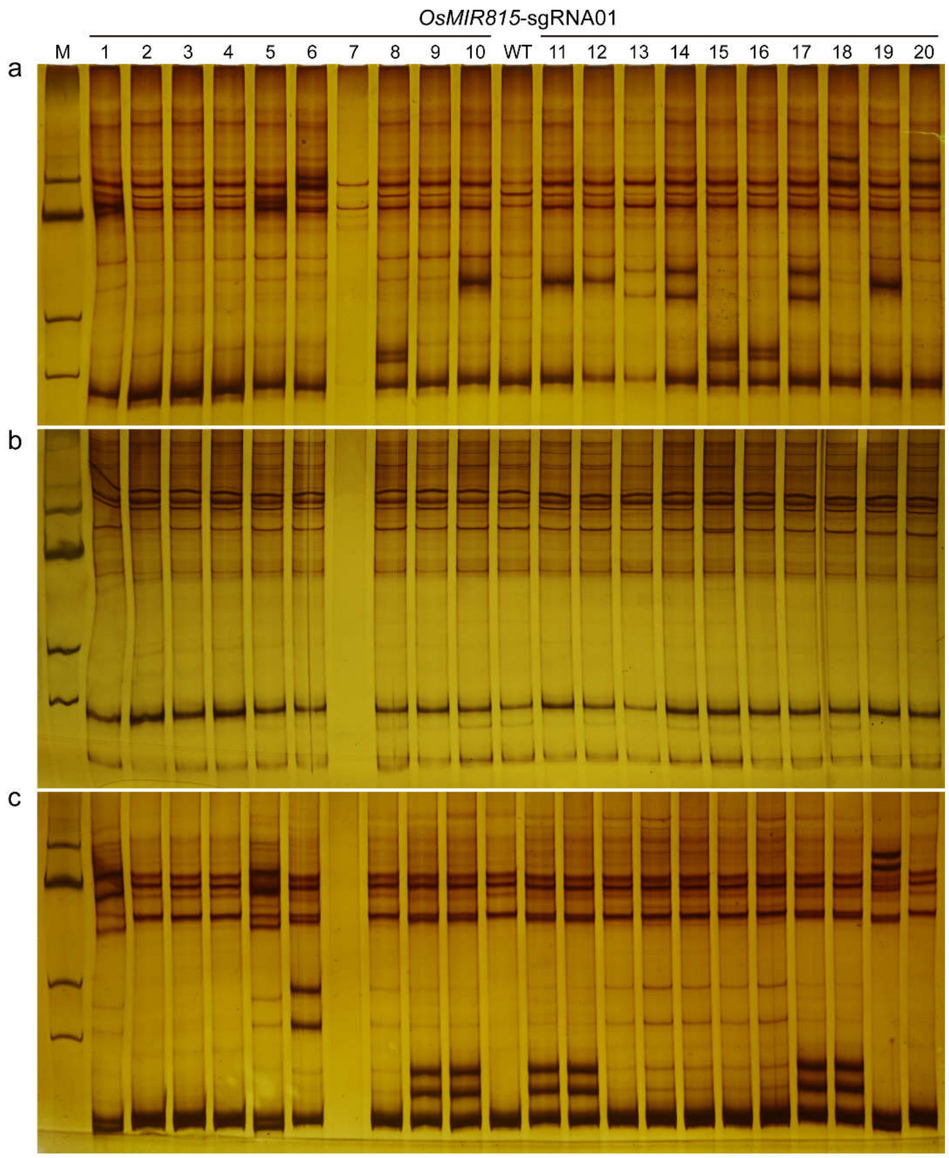

Supplementary Fig 8. Screen for genome-edited T0 lines targeted with *OsMIR815*-sgRNA01 by Sanger sequencing

```
#OsMIR815-sgRNA01-01
Ref. miR815a:    TGTGTTTGAGGAGAAGGGGATTGAGGAGATTGGGGAGATACGCAAAACGA
Allele 1:        TGTGTTTGAGGAGAAGGGGAT------GATTGGGGAGATACGCAAAACGA (-6  bp)
Allele 2:        TGTGTTTGAGGAGAAGGGGATTGAG--GATTGGGGAGATACGCAAAACGA (-2  bp)

Ref. miR815b:    GGGAGTGTTTGAGAAGGGGATTGAGGAGATTGGGAAGATACGTAAAACGA
Allele 1:        GGGAGTGTTTGAGAAGGGGAT-GAGGAGATTGGGAAGATACGTAAAACGA (-1  bp)
Allele 2:        GGGAGTGTTTGAGAAGGGGAT-GAGGAGATTGGGAAGATACGTAAAACGA (-1  bp)

Ref. miR815c:    TGTGTTTGAGGAGAAGGGGATTGAGGAGATTGGGAAGATACGCAAAACGA
Allele 1:        TGTGTTTGAGGAGAAGGGGAT----------GGGAAGATACGCAAAACGA (-10 bp)
Allele 2:        TGTGTTTGAGGAGAAGGGGATTGAGGAG-TTGGGAAGATACGCAAAACGA (-1  bp)

#OsMIR815-sgRNA01-05
Ref. miR815a:    TGTGTTTGAGGAGAAGGGGATTGAGGAGATTGGGGAGATACGCAAAACGA
Allele 1:        TGTGTTTGAGGAGAAGGGGAT------GATTGGGGAGATACGCAAAACGA (-6  bp)
Allele 2:        TGTGTTTGAGGAGAAGGGGATTGAG--GATTGGGGAGATACGCAAAACGA (-2  bp)

Ref. miR815b:    GGGAGTGTTTGAGAAGGGGATTGAGGAGATTGGGAAGATACGTAAAACGA
Allele 1:        GGGAGTGTTTGAGAAGGGGAT-GAGGAGATTGGGAAGATACGTAAAACGA (-1  bp)
Allele 2:        GGGAGTGTTTGAGAAGGGGAT-GAGGAGATTGGGAAGATACGTAAAACGA (-1  bp)

Ref. miR815c:    TGTGTTTGAGGAGAAGGGGATTGAGGAGATTGGGAAGATACGCAAAACGA
Allele 1:        TGTGTTTGAGGAGAAGGGGAT----------GGGAAGATACGCAAAACGA (-10 bp)
Allele 2:        TGTGTTTGAGGAGAAGGGGATTGAGGAG-TTGGGAAGATACGCAAAACGA (-1  bp)

#OsMIR815-sgRNA01-06
Ref. miR815a:    TGTGTTTGAGGAGAAGGGGATTGAGGAGATTGGGGAGATACGCAAAACGA
Allele 1:        TGTGTTTGAGGAGAAGGGGAT--------TTGGGGAGATACGCAAAACGA (-8  bp)
Allele 2:        TGTGTTTGAGGAGAAGGGGATTGAGGAGATTGGGGAGATACGCAAAACGA (WT)

Ref. miR815b:    GGGAGTGTTTGAGAAGGGGATTGAGGAGATTGGGAAGATACGTAAAACGA
Allele 1:        GGGAGTGTTTGAGAAGGGGATTGAGGAGA------AGATACGTAAAACGA (-6  bp)
Allele 2:        GGGAGTGTTTGAGAAGGGGATTGAGGAGATTGGGAAGATACGTAAAACGA (WT)

Ref. miR815c:    TGTGTTTGAGGAGAAGGGGATTGAGGAGATTGGGAAGATACGCAAAACGA
Allele 1:        TGTGTTTGAGGAGAAGGGGATTGAG---ATTGGGAAGATACGCAAAACGA (-3  bp)
Allele 2:        TGTGTTTGAGGAGAAGGGGATTGAGGAGATTGGGAAGATACGCAAAACGA (WT)

#OsMIR815-sgRNA01-08
Ref. miR815a:    TGTGTTTGAGGAGAAGGGGATTGAGGAGATTGGGGAGATACGCAAAACGA
Allele 1:        TGTGTTTGAGGAGAAGGGGATTGAGGA--TTGGGGAGATACGCAAAACGA (-2  bp)
Allele 2:        TGTGTTTGAGGAGAAGGGGATTGAGGAGATTGGGG-GATACGCAAAACGA (-1  bp)

Ref. miR815b:    GGGAGTGTTTGAGAAGGGGATTGAGGAGATTGGGAAGATACGTAAAACGA
Allele 1:        GGGAGTGTTTGAGAAGGGGAT-GAGGAGATTGGGAAGATACGTAAAACGA (-1  bp)
Allele 2:        GGGAGTGTTTGAGAAGGGGATTGAGGAGATTGGGAAGATACGTAAAACGA (WT)

Ref. miR815c:    TGTGTTTGAGGAGAAGGGGATTGAGGAGATTGGGAAGATACGCAAAACGA
Allele 1:        TGTGTTTGAGGAGAAGGGGATTGAGGAGATTGGGAAGATACGCAAAACGA (WT)
Allele 2:        TGTGTTTGAGGAGAAGGGGATTGAGGAGATTGGGAAGATACGCAAAACGA (WT)

#OsMIR815-sgRNA01-09
Ref. miR815a:    TGTGTTTGAGGAGAAGGGGATTGAGGAGATTGGGGAGATACGCAAAACGA
Allele 1:        TGTGTTTGAGGAGAAGGGGATTGAGGA--TTGGGGAGATACGCAAAACGA (-2  bp)
Allele 1:        TGTGTTTGAGGAGAAGGGGATTGAGGA--TTGGGGAGATACGCAAAACGA (-2  bp)

Ref. miR815b:    GGGAGTGTTTGAGAAGGGGATTGAGGAGATTGGGAAGATACGTAAAACGA
Allele 1:        GGGAGTGTTTGAGAAGGGGATTGAG---ATTGGGAAGATACGTAAAACGA (-3  bp)
Allele 2:        GGGAGTGTTTGAGAAGGGGATTGAGG--ATTGGGAAGATACGTAAAACGA (-2  bp)

Ref. miR815c:    TGTGTTTGAGGAGAAGGGGATTGAGGAGATTGGGAAGATACGCAAAACGA
Allele 1:        TGTGTTTGAGGAGAAGGGGATTGAGGA--TTGGGAAGATACGCAAAACGA (-2  bp)
Allele 2:        TGTGTTTGAGGAGAAGGGGATTGAGGAGATTGGGAAGATACGCAAAACGA (WT)
```

Supplementary Fig 8. Screen for genome-edited T0 lines targeted with *OsMIR815*-sgRNA01 by Sanger sequencing (continued)

```
#OsMIR815-sgRNA01-10
Ref. miR815a:    TGTGTTTGAGGAGAAGGGGATTGAGGAGATTGGGGAGATACGCAAAACGA
Allele 1:        TGTGTTTGAGGAGAAGGGGATTGA-----TTGGGGAGATACGCAAAACGA (-5  bp)
Allele 2:        TGTGTTTGAGGAGAAGGGGATTGA---GATTGGGGAGATACGCAAAACGA (-3  bp)

Ref. miR815b:    GGGAGTGTTTGAGAAGGGGATTGAGGAGATTGGGAAGATACGTAAAACGA
Allele 1:        GGGAGTGTTTGAGAAGGGGATTGAG---ATTGGGAAGATACGTAAAACGA (-3  bp)
Allele 2:        GGGAGTGTTTGAGAAGGGGATTGAGGAGATTGGGAAGATACGTAAAACGA (WT)

Ref. miR815c:    TGTGTTTGAGGAGAAGGGGATTGAGGAGATTGGGAAGATACGCAAAACGA
Allele 1:        TGTGTTTGAGGAGAAGGGGATTGAGGA--TTGGGAAGATACGCAAAACGA (-2  bp)
Allele 2:        TGTGTTTGAGGAGAAGGGGATTGAGGAGATTGGGAAGATACGCAAAACGA (WT)

#OsMIR815-sgRNA01-11
Ref. miR815a:    TGTGTTTGAGGAGAAGGGGATTGAGGAGATTGGGGAGATACGCAAAACGA
Allele 1:        TGTGTTTGAGGAGAAGGGGATTGA-----TTGGGGAGATACGCAAAACGA (-5  bp)
Allele 2:        TGTGTTTGAGGAGAAGGGGATTGA---GATTGGGGAGATACGCAAAACGA (-3  bp)

Ref. miR815b:    GGGAGTGTTTGAGAAGGGGATTGAGGAGATTGGGAAGATACGTAAAACGA
Allele 1:        GGGAGTGTTTGAGAAGGGGATTGAG---ATTGGGAAGATACGTAAAACGA (-3  bp)
Allele 2:        GGGAGTGTTTGAGAAGGGGATTGAGGAGATTGGGAAGATACGTAAAACGA (WT)

Ref. miR815c:    TGTGTTTGAGGAGAAGGGGATTGAGGAGATTGGGAAGATACGCAAAACGA
Allele 1:        TGTGTTTGAGGAGAAGGGGATTGAGGA--TTGGGAAGATACGCAAAACGA (-2  bp)
Allele 2:        TGTGTTTGAGGAGAAGGGGATTGAGGAGATTGGGAAGATACGCAAAACGA (WT)

#OsMIR815-sgRNA01-12
Ref. miR815a:    TGTGTTTGAGGAGAAGGGGATTGAGGAGATTGGGGAGATACGCAAAACGA
Allele 1:        TGTGTTTGAGGAGAAGGGGATTGA-----TTGGGGAGATACGCAAAACGA (-5  bp)
Allele 2:        TGTGTTTGAGGAGAAGGGGATTGA---GATTGGGGAGATACGCAAAACGA (-3  bp)

Ref. miR815b:    GGGAGTGTTTGAGAAGGGGATTGAGGAGATTGGGAAGATACGTAAAACGA
Allele 1:        GGGAGTGTTTGAGAAGGGGATTGAG---ATTGGGAAGATACGTAAAACGA (-3  bp)
Allele 2:        GGGAGTGTTTGAGAAGGGGATTGAGGAGATTGGGAAGATACGTAAAACGA (WT)

Ref. miR815c:    TGTGTTTGAGGAGAAGGGGATTGAGGAGATTGGGAAGATACGCAAAACGA
Allele 1:        TGTGTTTGAGGAGAAGGGGATTGAGGA--TTGGGAAGATACGCAAAACGA (-2  bp)
Allele 2:        TGTGTTTGAGGAGAAGGGGATTGAGGAGATTGGGAAGATACGCAAAACGA (WT)

#OsMIR815-sgRNA01-14
Ref. miR815a:    TGTGTTTGAGGAGAAGGGGATTGAGGAGATTGGGGAGATACGCAAAACGA
Allele 1:        TGTGTTTGAGGAGAAGGGGATTGAG---ATTGGGGAGATACGCAAAACGA (-3  bp)
Allele 2:        TGTGTTTGAGGAGAAGGGGATTGAGGAGATTGGGGAGATACGCAAAACGA (WT)

Ref. miR815b:    GGGAGTGTTTGAGAAGGGGATTGAGGAGATTGGGAAGATACGTAAAACGA
Allele 1:        GGGAGTGTTTGAGAAGGGGATTGAGGA--TTGGGAAGATACGTAAAACGA (-2  bp)
Allele 2:        GGGAGTGTTTGAGAAGGGGATTGAGGAGCTTGGGAAGATACGTAAAACGA (△1  bp)

Ref. miR815c:    TGTGTTTGAGGAGAAGGGGATTGAGGAGATTGGGAAGATACGCAAAACGA
Allele 1:        TGTGTTTGAGGAGAAGGGGATTGAGGAGATTGGGAAGATACGCAAAACGA (WT)
Allele 2:        TGTGTTTGAGGAGAAGGGGATTGAGGAGATTGGGAAGATACGCAAAACGA (WT)

#OsMIR815-sgRNA01-15
Ref. miR815a:    TGTGTTTGAGGAGAAGGGGATTGAGGAGATTGGGGAGATACGCAAAACGA
Allele 1:        TGTGTTTGAGGAGAAGGGGATTGAGGA--TTGGGGAGATACGCAAAACGA (-2  bp)
Allele 2:        TGTGTTTGAGGAGAAGGGGATTGAGGAGATTGGGG-GATACGCAAAACGA (-1  bp)

Ref. miR815b:    GGGAGTGTTTGAGAAGGGGATTGAGGAGATTGGGAAGATACGTAAAACGA
Allele 1:        GGGAGTGTTTGAGAAGGGGAT-GAGGAGATTGGGAAGATACGTAAAACGA (-1  bp)
Allele 2:        GGGAGTGTTTGAGAAGGGGATTGAGGAGATTGGGAAGATACGTAAAACGA (WT)

Ref. miR815c:    TGTGTTTGAGGAGAAGGGGATTGAGGAGATTGGGAAGATACGCAAAACGA
Allele 1:        TGTGTTTGAGGAGAAGGGGATTGAGGAGATTGGGAAGATACGCAAAACGA (WT)
Allele 2:        TGTGTTTGAGGAGAAGGGGATTGAGGAGATTGGGAAGATACGCAAAACGA (WT)
```

Supplementary Fig 8. Screen for genome-edited T0 lines targeted with *OsMIR815*-sgRNA01 by Sanger sequencing (continued)

```
#OsMIR815-sgRNA01-16
Ref. miR815a:    TGTGTTTGAGGAGAAGGGGATTGAGGAGATTGGGGAGATACGCAAAACGA
Allele 1:        TGTGTTTGAGGAGAAGGGGATTGAGGA--TTGGGGAGATACGCAAAACGA (-2  bp)
Allele 2:        TGTGTTTGAGGAGAAGGGGATTGAGGAGATTGGGG-GATACGCAAAACGA (-1  bp)

Ref. miR815b:    GGGAGTGTTTGAGAAGGGGATTGAGGAGATTGGGAAGATACGTAAAACGA
Allele 1:        GGGAGTGTTTGAGAAGGGGAT-GAGGAGATTGGGAAGATACGTAAAACGA (-1  bp)
Allele 2:        GGGAGTGTTTGAGAAGGGGATTGAGGAGATTGGGAAGATACGTAAAACGA (WT)

Ref. miR815c:    TGTGTTTGAGGAGAAGGGGATTGAGGAGATTGGGAAGATACGCAAAACGA
Allele 1:        TGTGTTTGAGGAGAAGGGGATTGAGGAGATTGGGAAGATACGCAAAACGA (WT)
Allele 2:        TGTGTTTGAGGAGAAGGGGATTGAGGAGATTGGGAAGATACGCAAAACGA (WT)

#OsMIR815-sgRNA01-17
Ref. miR815a:    TGTGTTTGAGGAGAAGGGGATTGAGGAGATTGGGGAGATACGCAAAACGA
Allele 1:        TGTGTTTGAGGAGAAGGGGATTGAG---ATTGGGGAGATACGCAAAACGA (-3  bp)
Allele 2:        TGTGTTTGAGGAGAAGGGGATTGAGGAGATTGGGGAGATACGCAAAACGA (WT)

Ref. miR815b:    GGGAGTGTTTGAGAAGGGGATTGAGGAGATTGGGAAGATACGTAAAACGA
Allele 1:        GGGAGTGTTTGAGAAGGGGATTGAGGA--TTGGGAAGATACGTAAAACGA (-2  bp)
Allele 2:        GGGAGTGTTTGAGAAGGGGATTGAGGAGCTTGGGAAGATACGTAAAACGA (△1  bp)

Ref. miR815c:    TGTGTTTGAGGAGAAGGGGATTGAGGAGATTGGGAAGATACGCAAAACGA
Allele 1:        TGTGTTTGAGGAGAAGGGGATTGAGGAGATTGGGAAGATACGCAAAACGA (WT)
Allele 2:        TGTGTTTGAGGAGAAGGGGATTGAGGAGATTGGGAAGATACGCAAAACGA (WT)

#OsMIR815-sgRNA01-18
Ref. miR815a:    TGTGTTTGAGGAGAAGGGGATTGAGGAGATTGGGGAGATACGCAAAACGA
Allele 1:        TGTGTTTGAGGAGAAGGGGATTGA-----TTGGGGAGATACGCAAAACGA (-5  bp)
Allele 2:        TGTGTTTGAGGAGAAGGGGATTGAGGAGATTGGGGAGATACGCAAAACGA (WT)

Ref. miR815b:    GGGAGTGTTTGAGAAGGGGATTGAGGAGATTGGGAAGATACGTAAAACGA
Allele 1:        GGGAGTGTTTGAGAAGGGGA---------TTGGGAAGATACGTAAAACGA (-9  bp)
Allele 2:        GGGAGTGTTTGAGAAGGGGATTGAG-AGATTGGGAAGATACGTAAAACGA (-1  bp)

Ref. miR815c:    TGTGTTTGAGGAGAAGGGGATTGAGGAGATTGGGAAGATACGCAAAACGA
Allele 1:        TGTGTTTGAGGAGAAGGGGATTGAGGA--TTGGGAAGATACGCAAAACGA (-2  bp)
Allele 2:        TGTGTTTGAGGAGAAGGGGATTGAGGAGATTGGGAAGATACGCAAAACGA (WT)

#OsMIR815-sgRNA01-19
Ref. miR815a:    TGTGTTTGAGGAGAAGGGGATTGAGGAGATTGGGGAGATACGCAAAACGA
Allele 1:        TGTGTTTGAGGAGAAGGGGATTGA-----TTGGGGAGATACGCAAAACGA (-5  bp)
Allele 2:        TGTGTTTGAGGAGAAGGGGATTGA---GATTGGGGAGATACGCAAAACGA (-3  bp)

Ref. miR815b:    GGGAGTGTTTGAGAAGGGGATTGAGGAGATTGGGAAGATACGTAAAACGA
Allele 1:        GGGAGTGTTTGAGAAGGGGATTGAG---ATTGGGAAGATACGTAAAACGA (-3  bp)
Allele 2:        GGGAGTGTTTGAGAAGGGGATTGAGGAGATTGGGAAGATACGTAAAACGA (WT)

Ref. miR815c:    TGTGTTTGAGGAGAAGGGGATTGAGGAGATTGGGAAGATACGCAAAACGA
Allele 1:        TGTGTTTGAGGAGAAGGGGATTGAGGA--TTGGGAAGATACGCAAAACGA (-2  bp)
Allele 2:        TGTGTTTGAGGAGAAGGGGATTGAGGAGATTGGGAAGATACGCAAAACGA (WT)

#OsMIR815-sgRNA01-20
Ref. miR815a:    TGTGTTTGAGGAGAAGGGGATTGAGGAGATTGGGGAGATACGCAAAACGA
Allele 1:        TGTGTTTGAGGAGAAGGGGATTGA-----TTGGGGAGATACGCAAAACGA (-5  bp)
Allele 2:        TGTGTTTGAGGAGAAGGGGATTGAGGAGATTGGGGAGATACGCAAAACGA (WT)

Ref. miR815b:    GGAGTGTTTGAGAAGGGGATTG-AGGAGATTGGGAAGATACGTAAAACGA
Allele 1:        GGAGTGTTTGAGAAGGGGA------GAGATTGGGAAGATACGTAAAACGA (-5  bp)
Allele 2:        GGAGTGTTTGAGAAGGGGATTGTAGGAGATTGGGAAGATACGTAAAACGA (+1  bp)

Ref. miR815c:    TGTGTTTGAGGAGAAGGGGATTGAGGAGATTGGGAAGATACGCAAAACGA
Allele 1:        TGTGTTTGAGGAGAAGGGGATTG--------GGGAAGATACGCAAAACGA (-8  bp)
Allele 2:        TGTGTTTGAGGAGAAGGGGATTGAG--GATTGGGAAGATACGCAAAACGA (-2  bp)
```

Supplementary Fig 9. Screen for genome-edited T0 lines targeted with *OsMIR820*-sgRNA01 by SSCP

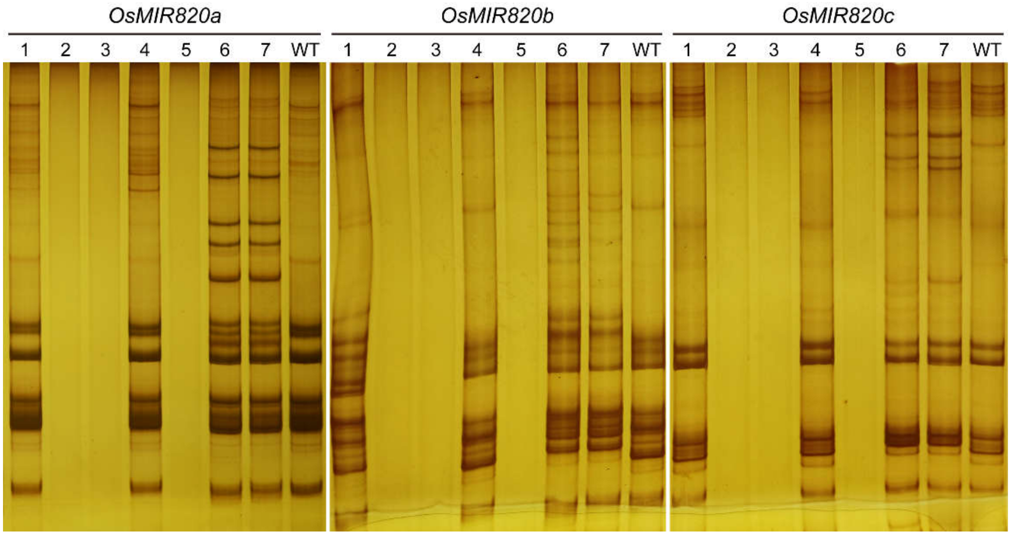

Supplementary Fig 10. Sanger sequencing of genome edited T0 lines targeted with *OsMIR820*-sgRNA01

```
#OsMIR820-sgRNA01-03-1
Ref. miR820a:    TCGTACATGCGTCGGCCTCGTGGATGG-ACCAGGAGCTCAACATTCCTTAA
Allele 1:        TCGTACATGCGTCGGCCTCGTGGATGGAACCAGGAGCTCAACATTCCTTAA (+1 bp)
Allele 2:        TCGTACATGCGTCGGCCTCGTGGATGGAACCAGGAGCTCAACATTCCTTAA (+1 bp)

Ref. miR820b:    TTGTACATGCGTCGGCCTCGTGGATGG-ACCAGGAGCTCGACGTTCCTTAA
Allele 1:        TTGTACATGCGTCGGCCTCGTGGAT---ACCAGGAGCTCGACGTTCCTTAA (-2 bp)
Allele 2:        TTGTACATGCGTCGGCCTCGTGGATGGTACCAGGAGCTCGACGTTCCTTAA (+1 bp)

Ref. miR820c:    TCGTACATGCGTCGGCCTCGTGGATGG-ACCAGGAGCTCGACATTCCTTAA
Allele 1:        TCGTACATGCGTCGGCCTCGTGGAT---ACCAGGAGCTCGACATTCCTTAA (-2 bp)
Allele 2:        TCGTACATGCGTCGGCCTCGTGGAT---ACCAGGAGCTCGACATTCCTTAA (-2 bp)

#OsMIR820-sgRNA01-03-2
Ref. miR820a:    TCGTACATGCGTCGGCCTCGTGGATGG-ACCAGGAGCTCAACATTCCTTAA
Allele 1:        TCGTACATGCGTCGGCCTCGTGGATGGAACCAGGAGCTCAACATTCCTTAA (+1 bp)
Allele 2:        TCGTACATGCGTCGGCCTCGTGGATGGAACCAGGAGCTCAACATTCCTTAA (+1 bp)

Ref. miR820b:    TTGTACATGCGTCGGCCTCGTGGATGG-ACCAGGAGCTCGACGTTCCTTAA
Allele 1:        TTGTACATGCGTCGGCCTCGTGGAT---ACCAGGAGCTCGACGTTCCTTAA (-2 bp)
Allele 2:        TTGTACATGCGTCGGCCTCGTGGATGGTACCAGGAGCTCGACGTTCCTTAA (+1 bp)

Ref. miR820c:    TCGTACATGCGTCGGCCTCGTGGATGG-ACCAGGAGCTCGACATTCCTTAA
Allele 1:        TCGTACATGCGTCGGCCTCGTGGAT---ACCAGGAGCTCGACATTCCTTAA (-2 bp)
Allele 2:        TCGTACATGCGTCGGCCTCGTGGAT---ACCAGGAGCTCGACATTCCTTAA (-2 bp)

#OsMIR820-sgRNA01-04-1
Ref. miR820a:    TCGTACATGCGTCGGCCTCGTGGATGG-ACCAGGAGCTCAACATTCCTTAA
Allele 1:        TCGTACATGCGTCGGCCTCGTGGAT---ACCAGGAGCTCAACATTCCTTAA (-2 bp)
Allele 2:        TCGTACATGCGTCGGCCTCGTGGATGGTACCAGGAGCTCAACATTCCTTAA (+1 bp)

Ref. miR820b:    TTGTACATGCGTCGGCCTCGTGGATGG-ACCAGGAGCTCGACGTTCCTTAA
Allele 1:        TTGTACATGCGTCGGCCTCGTGGAT---ACCAGGAGCTCGACGTTCCTTAA (-2 bp)
Allele 2:        TTGTACATGCGTCGGCCTCGTGGATGGTACCAGGAGCTCGACGTTCCTTAA (+1 bp)

Ref. miR820c:    TCGTACATGCGTCGGCCTCGTGGATGGA-CCAGGAGCTCGACATTCCTTAA
Allele 1:        TCGTACATGCGTCGGCCTCGTGGAT----CCAGGAGCTCGACATTCCTTAA (-3 bp)
Allele 2:        TCGTACATGCGTCGGCCTCGTGGAT-GA-CCAGGAGCTCGACATTCCTTAA (-1 bp)

#OsMIR820-sgRNA01-04-2
Ref. miR820a:    TCGTACATGCGTCGGCCTCGTGGATGG-ACCAGGAGCTCAACATTCCTTAA
Allele 1:        TCGTACATGCGTCGGCCTCGTGGAT---ACCAGGAGCTCAACATTCCTTAA (-2 bp)
Allele 2:        TCGTACATGCGTCGGCCTCGTGGATGGTACCAGGAGCTCAACATTCCTTAA (+1 bp)

Ref. miR820b:    TTGTACATGCGTCGGCCTCGTGGATGG-ACCAGGAGCTCGACGTTCCTTAA
Allele 1:        TTGTACATGCGTCGGCCTCGTGGAT---ACCAGGAGCTCGACGTTCCTTAA (-2 bp)
Allele 2:        TTGTACATGCGTCGGCCTCGTGGATGGTACCAGGAGCTCGACGTTCCTTAA (+1 bp)

Ref. miR820c:    TCGTACATGCGTCGGCCTCGTGGATGGA-CCAGGAGCTCGACATTCCTTAA
Allele 1:        TCGTACATGCGTCGGCCTCGTGGAT----CCAGGAGCTCGACATTCCTTAA (-3 bp)
Allele 2:        TCGTACATGCGTCGGCCTCGTGGAT-GA-CCAGGAGCTCGACATTCCTTAA (-1 bp)

#OsMIR820-sgRNA01-04-3
Ref. miR820a:    TCGTACATGCGTCGGCCTCGTGGATGG-ACCAGGAGCTCAACATTCCTTAA
Allele 1:        TCGTACATGCGTCGGCCTCGTGGAT---ACCAGGAGCTCAACATTCCTTAA (-2 bp)
Allele 2:        TCGTACATGCGTCGGCCTCGTGGATGGTACCAGGAGCTCAACATTCCTTAA (+1 bp)

Ref. miR820b:    TTGTACATGCGTCGGCCTCGTGGATGG-ACCAGGAGCTCGACGTTCCTTAA
Allele 1:        TTGTACATGCGTCGGCCTCGTGGAT---ACCAGGAGCTCGACGTTCCTTAA (-2 bp)
Allele 2:        TTGTACATGCGTCGGCCTCGTGGATGGTACCAGGAGCTCGACGTTCCTTAA (+1 bp)

Ref. miR820c:    TCGTACATGCGTCGGCCTCGTGGATGGA-CCAGGAGCTCGACATTCCTTAA
Allele 1:        TCGTACATGCGTCGGCCTCGTGGAT----CCAGGAGCTCGACATTCCTTAA (-3 bp)
Allele 2:        TCGTACATGCGTCGGCCTCGTGGAT-GA-CCAGGAGCTCGACATTCCTTAA (-1 bp)
```

Supplementary Fig 10. Sanger sequencing of genome edited T0 lines targeted with *OsMIR820*-sgRNA01 (continued)

```
#OsMIR820-sgRNA01-04-4
Ref. miR820a:    TCGTACATGCGTCGGCCTCGTGGATGG-ACCAGGAGCTCAACATTCCTTAA
Allele 1:        TCGTACATGCGTCGGCCTCGTGGAT---ACCAGGAGCTCAACATTCCTTAA (-2  bp)
Allele 2:        TCGTACATGCGTCGGCCTCGTGGATGGTACCAGGAGCTCAACATTCCTTAA (+1  bp)

Ref. miR820b:    TTGTACATGCGTCGGCCTCGTGGATGG-ACCAGGAGCTCGACGTTCCTTAA
Allele 1:        TTGTACATGCGTCGGCCTCGTGGAT---ACCAGGAGCTCGACGTTCCTTAA (-2  bp)
Allele 2:        TTGTACATGCGTCGGCCTCGTGGATGGTACCAGGAGCTCGACGTTCCTTAA (+1  bp)

Ref. miR820c:    TCGTACATGCGTCGGCCTCGTGGATGGA-CCAGGAGCTCGACATTCCTTAA
Allele 1:        TCGTACATGCGTCGGCCTCGTGGAT----CCAGGAGCTCGACATTCCTTAA (-3  bp)
Allele 2:        TCGTACATGCGTCGGCCTCGTGGAT-GA-CCAGGAGCTCGACATTCCTTAA (-1  bp)

#OsMIR820-sgRNA01-06
Ref. miR820a:    TCGTACATGCGTCGGCCTCGTGGATGG-ACCAGGAGCTCAACATTCCTTAA
Allele 1:        TCGTACATGCGTCGGCCTCGTGGATGGGACCAGGAGCTCAACATTCCTTAA (+1  bp)
Allele 2:        TCGTACATGCGTCGGCCTCGTGGA-----CCAGGAGCTCAACATTCCTTAA (-4  bp)

Ref. miR820b:    TTGTACATGCGTCGGCCTCGTGGAT-GGACCAGGAGCTCGACGTTCCTTAA
Allele 1:        TTGTACATGCGTCGGCCTCGTGGAT--TACCAGGAGCTCGACGTTCCTTAA (-1bp/△1bp)
Allele 2:        TTGTACATGCGTCGGCCTCGTGGATAGGACCAGGAGCTCGACGTTCCTTAA (+1  bp)

Ref. miR820c:    TCGTACATGCGTCGGCCTCGTGGATGG-ACCAGGAGCTCGACATTCCTTAA
Allele 1:        TCGTACATGCGTCGGCCTCGTGGATGGAACCAGGAGCTCGACATTCCTTAA (+1  bp)
Allele 2:        TCGTACATGCGTCGGCCTCGTGGATGGAACCAGGAGCTCGACATTCCTTAA (+1  bp)

#OsMIR820-sgRNA01-07
Ref. miR820a:    TCGTACATGCGTCGGCCTCGTGGATGG-ACCAGGAGCTCAACATTCCTTAA
Allele 1:        TCGTACATGCGTCGGCCTCGTGGATGGGACCAGGAGCTCAACATTCCTTAA (+1  bp)
Allele 2:        TCGTACATGCGTCGGCCTCGTGGA-----CCAGGAGCTCAACATTCCTTAA (-4  bp)

Ref. miR820b:    TTGTACATGCGTCGGCCTCGTGGAT-GGACCAGGAGCTCGACGTTCCTTAA
Allele 1:        TTGTACATGCGTCGGCCTCGTGGAT--TACCAGGAGCTCGACGTTCCTTAA (-1bp/△1bp)
Allele 2:        TTGTACATGCGTCGGCCTCGTGGATAGGACCAGGAGCTCGACGTTCCTTAA (+1  bp)

Ref. miR820c:    TCGTACATGCGTCGGCCTCGTGGATGG-ACCAGGAGCTCGACATTCCTTAA
Allele 1:        TCGTACATGCGTCGGCCTCGTGGATGGAACCAGGAGCTCGACATTCCTTAA (+1  bp)
Allele 2:        TCGTACATGCGTCGGCCTCGTGGATGGAACCAGGAGCTCGACATTCCTTAA (+1  bp)

#OsMIR820-sgRNA01-09-01
Ref. miR820a:    TCGTACATGCGTCGGCCTCGTGGATGGACCAGGAGCTCAACATTCCTTAA
Allele 1:        TCGTACATGCGTCGGCCTCGT-------CCAGGAGCTCAACATTCCTTAA  (-7  bp)
Allele 1:        TCGTACATGCGTCGGCCTCGTGGATGGACCAGGAGCTCAACATTCCTTAA  (WT)

Ref. miR820b:    TTGTACATGCGTCGGCCTCGTGGATGGACCAGGAGCTCGACGTTCCTTAA
Allele 1:        TTGTACATGCGTCGGCCTCGTCCA----CCAGGAGCTCGACGTTCCTTAA  (-4  bp)
Allele 2:        TTGTACATGCGTCGGCCTCGTGGA-CGACCAGGAGCTCGACGTTCCTTAA  (-1bp/△1bp)

Ref. miR820c:    TCGTACATGCGTCGGCCTCGTGGATGGACCAGGAGCTCGACATTCCTTAA
Allele 1:        TCGTACATGCGTCGGCCTCGTGGATG-ACCAGGAGCTCGACATTCCTTAA  (-1  bp)
Allele 2:        TCGTACATGCGTCGGCCTCGTGGATGGACCAGGAGCTCGACATTCCTTAA  (WT)
```

Supplementary Fig 10. Sanger sequencing of genome edited T0 lines targeted with *OsMIR820*-sgRNA01 (continued)

```
#OsMIR820-sgRNA01-09-02
Ref. miR820a:    TCGTACATGCGTCGGCCTCGTGGATGGACCAGGAGCTCAACATTCCTTAA
Allele 1:        TCGTACATGCGTCGGCCTCGT-------CCAGGAGCTCAACATTCCTTAA  (-7  bp)
Allele 1:        TCGTACATGCGTCGGCCTCGTGGATGGACCAGGAGCTCAACATTCCTTAA  (WT)

Ref. miR820b:    TTGTACATGCGTCGGCCTCGTGGATGGACCAGGAGCTCGACGTTCCTTAA
Allele 1:        TTGTACATGCGTCGGCCTCGTCCA----CCAGGAGCTCGACGTTCCTTAA  (-4  bp)
Allele 2:        TTGTACATGCGTCGGCCTCGTGGA-CGACCAGGAGCTCGACGTTCCTTAA  (-1bp/△1bp)

Ref. miR820c:    TCGTACATGCGTCGGCCTCGTGGATGGACCAGGAGCTCGACATTCCTTAA
Allele 1:        TCGTACATGCGTCGGCCTCGTGGATG-ACCAGGAGCTCGACATTCCTTAA  (-1  bp)
Allele 2:        TCGTACATGCGTCGGCCTCGTGGATGGACCAGGAGCTCGACATTCCTTAA  (WT)

#OsMIR820-sgRNA01-10-01
Ref. miR820a:    TCGTACATGCGTCGGCCTCGTGGATGGACCAGGAGCTCAACATTCCTTAA
Allele 1:        TCGTACATGCGTCGGCCTCGTGGATGGACCAGGAGCTCAACATTCCTTAA  (WT)
Allele 2:        TCGTACATGCGTCGGCCTCGTGGATGGACCAGGAGCTCAACATTCCTTAA  (WT)

Ref. miR820b:    TTGTACATGCGTCGGCCTCGTGGATGG-ACCAGGAGCTCGACGTTCCTTAA
Allele 1:        TTGTACATGCGTCGGCCTCGTGGATG--ACCAGGAGCTCGACGTTCCTTAA (-1  bp)
Allele 2:        TTGTACATGCGTCGGCCTCGTGGATGGTACCAGGAGCTCGACGTTCCTTAA (+1  bp)

Ref. miR820c:    TCGTACATGCGTCGGCCTCGTGGATGGACCAGGAGCTCGACATTCCTTAA
Allele 1:        TCGTACATGCGTCGGCCTCGTGGATGGACCAGGAGCTCGACATTCCTTAA  (WT)
Allele 2:        TCGTACATGCGTCGGCCTCGTGGATGGACCAGGAGCTCGACATTCCTTAA  (WT)

#OsMIR820-sgRNA01-10-02
Ref. miR820a:    TCGTACATGCGTCGGCCTCGTGGATGGACCAGGAGCTCAACATTCCTTAA
Allele 1:        TCGTACATGCGTCGGCCTCGTGGATGGTCCAGGAGCTCAACATTCCTTAA  (△1  bp)
Allele 2:        TCGTACATGCGTCGGCCTCGTGGATGGACCAGGAGCTCAACATTCCTTAA  (WT)

Ref. miR820b:    TTGTACATGCGTCGGCCTCGTGGATGGACCAGGAGCTCGACGTTCCTTAA
Allele 1:        TTGTACATGCGTCGGCCTCGTGGATG-ACCAGGAGCTCGACGTTCCTTAA  (-1  bp)
Allele 2:        TTGTACATGCGTCGGCCTCGTGGATGGACCAGGAGCTCGACGTTCCTTAA  (WT)

Ref. miR820c:    TCGTACATGCGTCGGCCTCGTGGATGGACCAGGAGCTCGACATTCCTTAA
Allele 1:        TCGTACATGCGTCGGCCTCGTGGATGGACCAGGAGCTCGACATTCCTTAA  (WT)
Allele 2:        TCGTACATGCGTCGGCCTCGTGGATGGACCAGGAGCTCGACATTCCTTAA  (WT)

#OsMIR820-sgRNA01-10-03
Ref. miR820a:    TCGTACATGCGTCGGCCTCGTGGATGGACCAGGAGCTCAACATTCCTTAA
Allele 1:        T---------------------------CCAGGAGCTCAACATTCCTTAA  (-28 bp)
Allele 2:        T---------------------------CCAGGAGCTCAACATTCCTTAA  (-28 bp)

Ref. miR820b:    TTGTACATGCGTCGGCCTCGTGGATGGACCAGGAGCTCGACGTTCCTTAA
Allele 1:        TTGTACATGCGTCGGCCTCGTGGAT----CAGGAGCTCGACGTTCCTTAA  (-4  bp)
Allele 2:        TTGTACATGCGTCGGCCTCGTGGAT----CAGGAGCTCGACGTTCCTTAA  (-4  bp)

Ref. miR820c:    TCGTACATGCGTCGGCCTCGTGGATGGACCAGGAGCTCGACATTCCTTAA
Allele 1:        TCGTACATGCGTCGGCCTCGTGGAT--ACCAGGAGCTCGACATTCCTTAA  (-2  bp)
Allele 2:        TCGTACATGCGTCGGCCTCGTGGAT--ACCAGGAGCTCGACATTCCTTAA  (-2  bp)

#OsMIR820-sgRNA01-10-04
Ref. miR820a:    TCGTACATGCGTCGGCCTCGTGGATGG-ACCAGGAGCTCAACATTCCTTAA
Allele 1:        TCGTACATGCGTCGGCCTCGTGGATGGAACCAGGAGCTCAACATTCCTTAA (+1  bp)
Allele 1:        TCGTACATGCGTCGGCCTCGTGGATGGAACCAGGAGCTCAACATTCCTTAA (+1  bp)

Ref. miR820b:    TTGTACATGCGTCGGCCTCGTGGATGG-ACCAGGAGCTCGACGTTCCTTAA
Allele 1:        TTGTACATGCGTCGGCCTCGTGGA-----CCAGGAGCTCGACGTTCCTTAA (-4  bp)
Allele 2:        TTGTACATGCGTCGGCCTCGTGGATGGCACCAGGAGCTCGACGTTCCTTAA (+1  bp)

Ref. miR820c:    TCGTACATGCGTCGGCCTCGTGGATGGACCAGGAGCTCGACATTCCTTAA
Allele 1:        TCGTACATGCGTCGGCCTCGTGGAT--ACCAGGAGCTCGACATTCCTTAA  (-2  bp)
Allele 2:        TCGTACATGCGTCGGCCTCGTGGATGG---AGGAGCTCGACATTCCTTAA  (-3  bp)
```

Supplementary Fig 11. Phenotype of *OsMIR528* mutant under salt stress

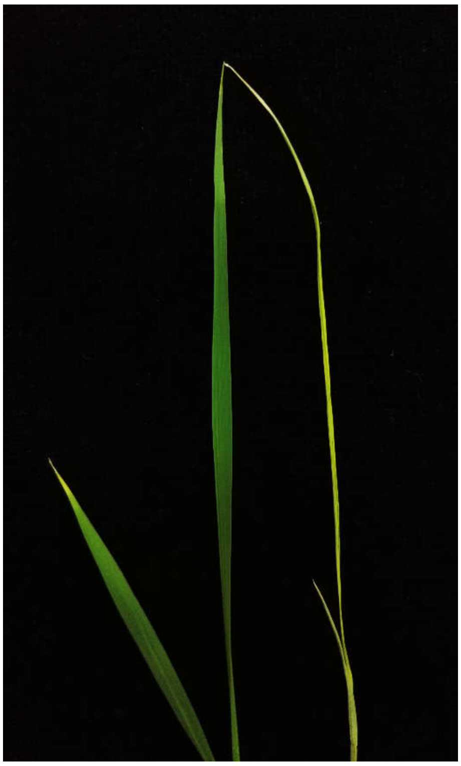

WT Mutant

Supplementary Fig 12. De-repression of a putative target gene of *OsMIR408* in the *OsMIR408* mutant background

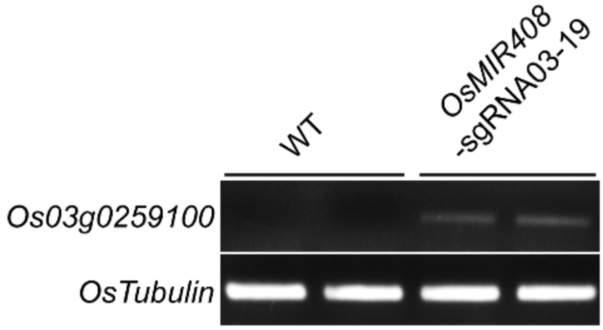

Supplementary Fig 13. 1bp indels in mature OsMIR528 don't affect the expression of putative target genes.

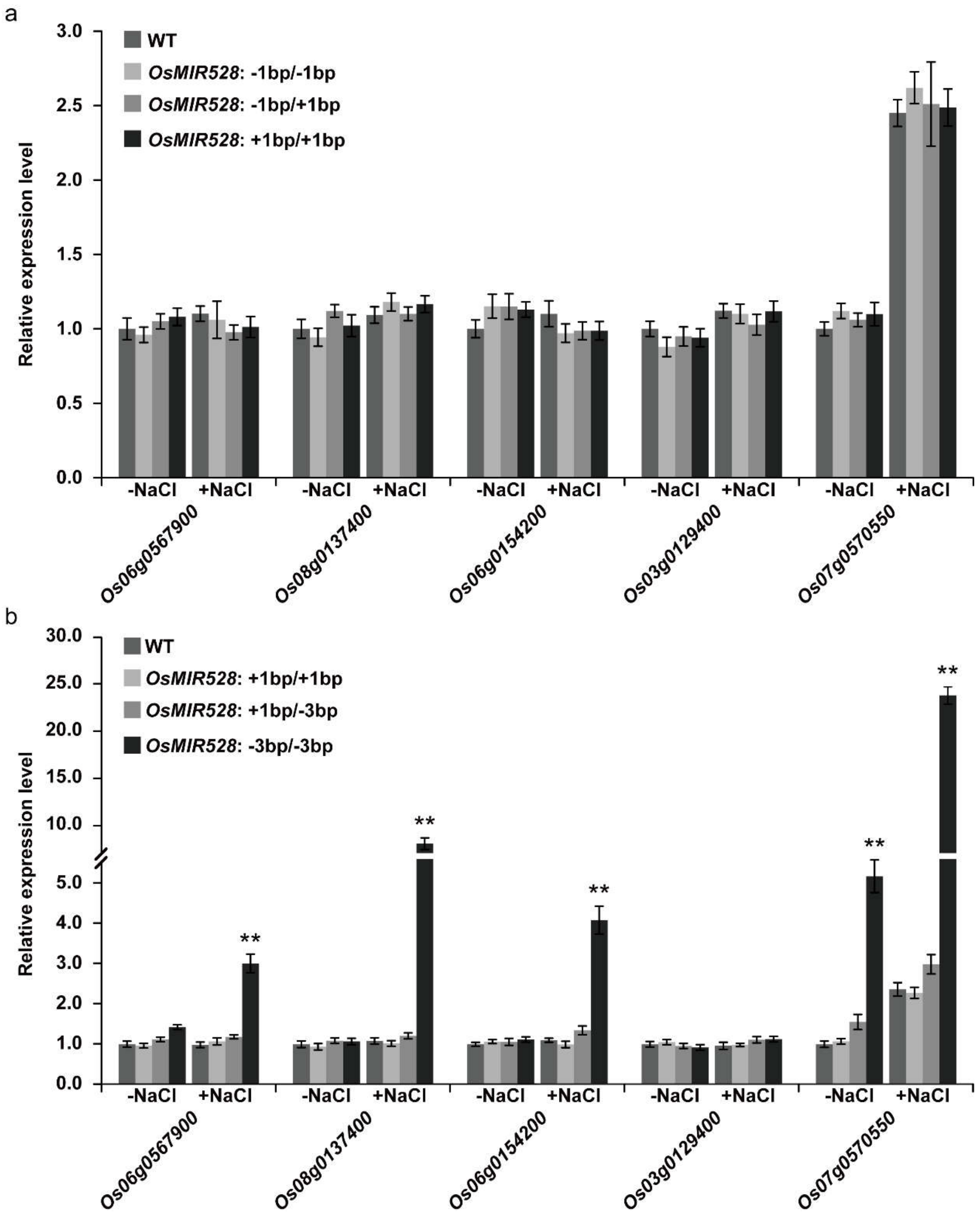

Supplementary Fig 14. Targeted deletion of *OsMIR408* and *OsMIR528* loci in rice protoplasts

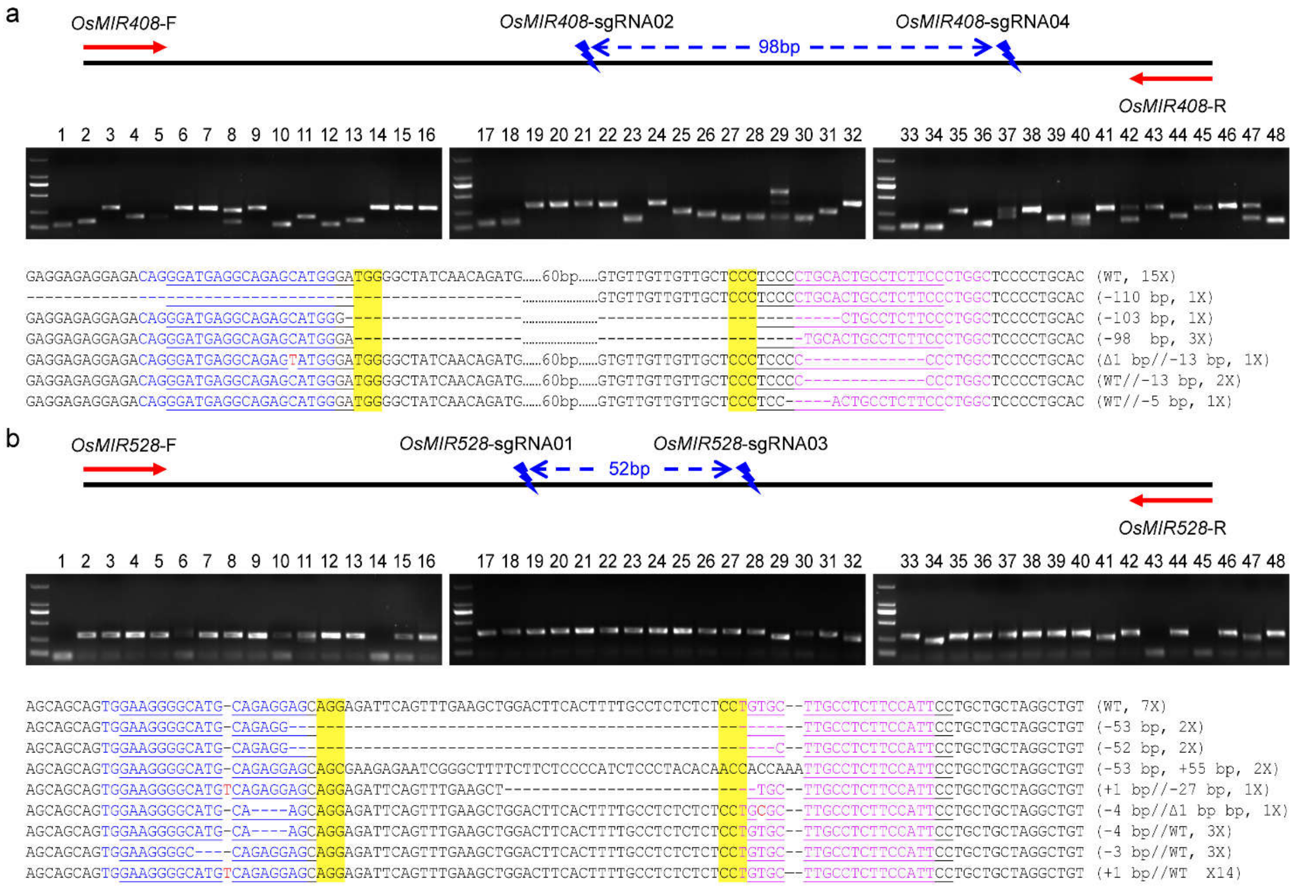

c

Targeted rice pri-microRNA locus	CRISPR/Cas9 vector	Sequenced T-A single clones	Deletion amplicons: number, ratio	Double NHEJ amplicons: number, ratio	Single NHEJ amplicons: number, ratio
OsMIR408	*pZmUbi1::Cas9+OsU6:: OsMIR408-sgRNA02+gRNA04*	24	5, 20.8%	1, 4.2%	3, 12.5%
OsMIR528	*pZmUbi1::Cas9+OsU6:: OsMIR528-sgRNA01+gRNA03*	35	6, 17.1%	2, 5.7%	20, 57.1%

Supplementary Fig 15. Generation of large deletion T0 mutants at pri-miR408 and pri-miR528 loci with two simultaneous DNA DSBs

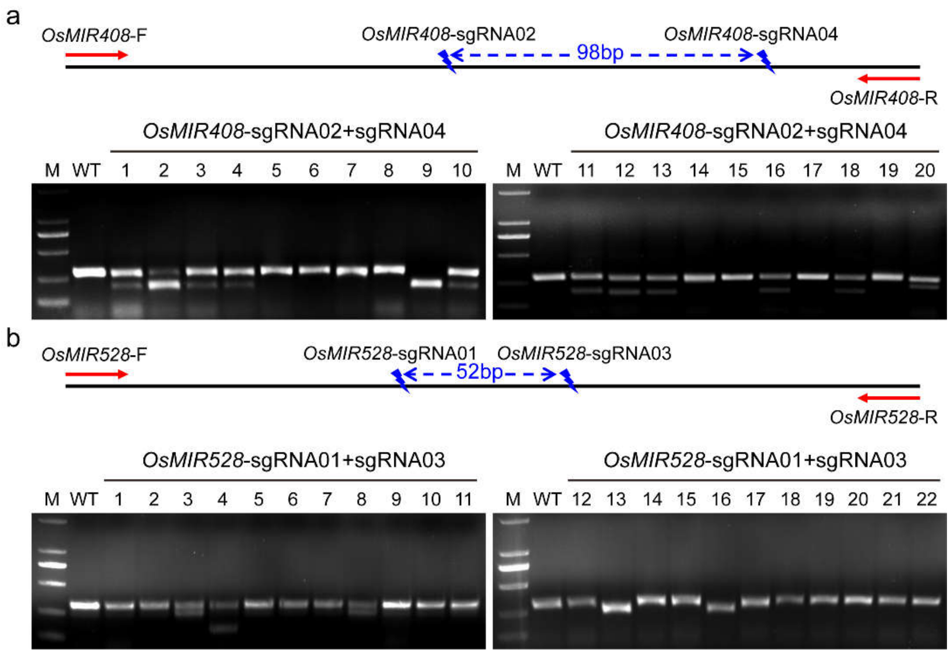

Supplementary Fig 16. Genotyping rice T0 lines for deletions at *OsMIR408* Allele

pZmUbi1::Cas9+OsU6::OsMIR*408*-sgRNA02+sgRNA04 (pZJP025)

```
Reference:                GAGGAGAGGAGACAGGGATGAGGCAGAGCATGGGA-TGGGGCTATCAACAGATGTAG......55bp......TGGTGTTGTTGTTGCTCCCTC-CCCTGCACTGCCTCTTCCCTGGCTCCCCTGCAC
#OsMIR408-sgRNA02+sgRNA04-01
Allele-1:                 GAGGAGAGGAGACAGGGATGAGGCAGAGCATGGG----------------------------------------------------CCTGCACTGCCTCTTCCCTGGCTCCCCTGCAC (-99 bp
Allele-2:                 GAGGAGAGGAGACAGGGATGAGGCAGAGCATGGG------GCTATCAACAGATGTAG......55bp......TGGTGTTGTTGTTGCTCCCTC------CACTGCCTCTTCCCTGGCTCCCCTGCAC (-5 bp//-5 bp)
#OsMIR408-sgRNA02+sgRNA04-02
Allele-1:                 GAGGAGAGGAGACAGGGATGAGGCAGAGCATGGGA-TGGG---------------------------------------------------ACTGCCTCTTCCCTGGCTCCCCTGCAC (-99 bp)
Allele-2:                 GAGGAGAGGAGACAGGGATGAGGCAGAGCATGG---TGGGGCTATCAACAGATGTAG......55bp......TGGTGTTGTTGTTGCTCCCTCTCCCTGCACTGCCTCTTCCCTGGCTCCCCTGCAC (-2 bp//+1 bp)
#OsMIR408-sgRNA02+sgRNA04-03
Allele-1:                 GAGGAGAGGAGACAGGGATGAGGCAGAGCATGGG----------------------------------------------------CCTGCACTGCCTCTTCCCTGGCTCCCCTGCAC (-99 bp)
Allele-2:                 GAGGAGAGGAGACAGGGATGAGGCAGAGCATGGGAATGGGGCTATCAACAGATGTAG......55bp......TGGTGTTGTTGTTGCTCCCTC---CTG-ACTGCCTCTTCCCTGGCTCCCCTGCAC (+1 bp//-3 bp)
#OsMIR408-sgRNA02+sgRNA04-04
ocus-1:                   GAGGAGAGGAGACAGGGATGAGGCAGAGCATGGG----------------------------------------------------CCTGCACTGCCTCTTCCCTGGCTCCCCTGCAC (-99 bp)
Allele-2:                 GAGGAGAGGAGACAGGGATGAGGCAGAGCATGGG--TGGGGCTATCAACAGATGTAG......55bp......TGGTGTTGTTGTTGCTCCCTC---CTG-ACTGCCTCTTCCCTGGCTCCCCTGCAC (-1 bp//-3 bp)
#OsMIR408-sgRNA02+sgRNA04-05
Allele-1:                 GAGGAGAGGAGACAGGGATGAGGCAGAGCATGGGAATGGGGCTATCAACAGATGTAG......55bp......TGGTGTTGTTGTTGCTCCCTC-CC-TGCACTGCCTCTTCCCTGGCTCCCCTGCAC (+1 bp//-1 bp)
Allele-2:                 GAGGAGAGGAGACAGGGATGAGGCAGAGCATGGGTATGGGGCTATCAACAGATGTAG......55bp......TGGTGTTGTTGTTGCTCCCTC-CC-TGCACTGCCTCTTCCCTGGCTCCCCTGCAC (+1 bp//-1 bp)
#OsMIR408-sgRNA02+sgRNA04-06
Allele-1:                 GAGGAGAGGAGACAGGGATGAGGCAGAGCATGGGAATGGGGCTATCAACAGATGTAG......55bp......TGGTGTTGTTGTTGCTCCCTCCCCCTGCACTGCCTCTTCCCTGGCTCCCCTGCAC (+1 bp//+1 bp)
Allele-2:                 GAGGAGAGGAGACAGGGATGAGGCAGA--ATGGGA-TGGGGCTATCAACAGATGTAG......55bp......TGGTGTTGTTGTTGCTCCCTCCCCCTGCACTGCCTCTTCCCTGGCTCCCCTGCAC (-2 bp//+1 bp)
#OsMIR408-sgRNA02+sgRNA04-07
Allele-1:                 GAGGAGAGGAGACAGGGATGAGGCAGAGCA----A-TGGGGCTATCAACAGATGTAG......55bp......TGGTGTTGTTGTTGCTCCCTCCCCCTGCACTGCCTCTTCCCTGGCTCCCCTGCAC (-4  bp//+1 bp)
Allele-2:                 GAGGAGAGGAGACAGGGATGAGGCAGAGCATGGGA-T--------------------......52bp......TGGTGTTGTTGTTGCTCCCTC-CC-T--ACTGCCTCTTCCCTGGCTCCCCTGCAC (-23 bp//-3 bp)
#OsMIR408-sgRNA02+sgRNA04-08
Allele-1:                 GAGGAGAGGAGACAGGGATGAGGCAGAGCA----A-TGGGGCTATCAACAGATGTAG......55bp......TGGTGTTGTTGTTGCTCCCTCCCCCTGCACTGCCTCTTCCCTGGCTCCCCTGCAC (-4  bp//+1 bp)
Allele-2:                 GAGGAGAGGAGACAGGGATGAGGCAGAGCATGGGA-T--------------------......52bp......TGGTGTTGTTGTTGCTCCCTC-CC-T--ACTGCCTCTTCCCTGGCTCCCCTGCAC (-23 bp//-3 bp)
#OsMIR408-sgRNA02+sgRNA04-09
Allele-1:                 GAGGAGAGGAGACAGGGATGAGGCAGAGCA---------------------------------------------------------------------CCCTGGCTCCCCTGCAC (-117 bp)
Allele-1:                 GAGGAGAGGAGACAGGGATGAGGCAGAGCA---------------------------------------------------------------------CCCTGGCTCCCCTGCAC (-117 bp)
#OsMIR408-sgRNA02+sgRNA04-10
Allele-1:                 GAGGAGAGGAGACAGGGATGAGGCAGAGCATGGG----------------------------------------------------CCTGCACTGCCTCTTCCCTGGCTCCCCTGCAC (-99  bp)
Allele-2:                 GAGGAGAGGAGACAGGGATGAGGCAGAGCATGGG--TGGGGCTATCAACAGATGTAG......55bp......TGGTGTTGTTGTTGCTCCCTC---CTG-ACTGCCTCTTCCCTGGCTCCCCTGCAC (-1  bp//-3 bp)
#OsMIR408-sgRNA02+sgRNA04-11
Allele-1:                 GAGGAGAGGAGACAGGGATGAGGCAGAGCATGGG---------------------------------------------CCCTC-CCCTGCACTGCCTCTTCCCTGGCTCCCCTGCAC (-92  bp)
Allele-2:                 GAGGAGAGGAGACAGGGATGAGGCAGAGCATGGGG-GAGGGAGCAACAACAACACCA......55bp......CTACATCTGTTGATAGCCCCA-TCCTGCACTGCCTCTTCCCTGGCTCCCCTGCAC (99  bp inversion)
#OsMIR408-sgRNA02+sgRNA04-12
Allele-1:                 GAGGAGAGGAGACAGGGATGAGGCAGA---------TGGGGCTATCAACAGATGTAG......55bp......TGGTGTTGTTGTTGCTCCCTC-C--TGCACTGCCTCTTCCCTGGCTCCCCTGCAC (-8  bp//-2 bp)
Allele-2:                 GAGGAGAGGAGACAGGGATGAGGCAGAGC------------------------------------------------------------CTGCCTCTTCCCTGGCTCCCCTGCAC (110 bp)
#OsMIR408-sgRNA02+sgRNA04-13
Allele-1:                 GAGGAGAGGAGACAGGGATGAGGCAGA---------TGGGGCTATCAACAGATGTAG......55bp......TGGTGTTGTTGTTGCTCCCTC-C--TGCACTGCCTCTTCCCTGGCTCCCCTGCAC (-8  bp//-2 bp)
Allele-2:                 GAGGAGAGGAGACAGGGATGAGGCAGAGC------------------------------------------------------------CTGCCTCTTCCCTGGCTCCCCTGCAC (110 bp)
#OsMIR408-sgRNA02+sgRNA04-14
Allele-1:                 GAGGAGAGGAGACAGGGATG------------------GGGCTATCAACAGATGTAG......55bp......TGGTGTTGTTGTTGCTCCCTC-C---GCACTGCCTCTTCCCTGGCTCCCCTGCAC (-17 bp//-3 bp)
Allele-2:                 GAGGAGAGGAGACAGGGATGAGGCAGAGCAT-----TGGGGCTATCAACAGATGTAG......55bp......TGGTGTTGTTGTTGCTCCCTC---------TGCCTCTTCCCTGGCTCCCCTGCAC (-5  bp//-8 bp)
#OsMIR408-sgRNA02+sgRNA04-15
Allele-1:                 GAGGAGAGGAGACAGGGATGAGGCAGAGCAT---A-TGGGGCTATCAACAGATGTAG......55bp......TGGTGTTGTTGTTGCTCCCTC-C-CTGCACTGCCTCTTCCCTGGCTCCCCTGCAC (-3  bp//-1 bp)
Allele-2:                 GAGGAGAGGAGACAGGGATGAGGCAGAGCAT---A-TGGGGCTATCAACAGATGTAG......55bp......TGGTGTTGTTGTTGCTCCCTC-C-CTGCACTGCCTCTTCCCTGGCTCCCCTGCAC (-3  bp//-1 bp)
#OsMIR408-sgRNA02+sgRNA04-16
Allele-1:                 GAGGAGAGGAGACAGGGATGAGGCAGAGCATGGG----------------------------------------------------CCTGCACTGCCTCTTCCCTGGCTCCCCTGCAC (-99  bp)
Allele-2:                 GAGGAGAGGAGACAGGGATGAGGCAGAGCATGG-A-TGGGGCTATCAACAGATGTAG......55bp......TGGTGTTGTTGTTGCTCCCTC-C-CCTGACTGCCTCTTCCCTGGCTCCCCTGCAC (-1  bp//-1 bp)
#OsMIR408-sgRNA02+sgRNA04-17
Allele-1:                 GAGGAGAGGAGACAGGGATGAGGCAGAGCATGGGGATGGGGCTATCAACAGATGTAG......55bp......TGGTGTTGTTGTTGCTCCCTC-CC-TGCACTGCCTCTTCCCTGGCTCCCCTGCAC (+1  bp//-1 bp)
Allele-2:                 GAGGAGAGGAGACAGGGATGAGGCAGAGCATGGGGATGGGGCTATCAACAGATGTAG......55bp......TGGTGTTGTTGTTGCTCCCTC-CC-TGCACTGCCTCTTCCCTGGCTCCCCTGCAC (+1  bp//-1 bp)
#OsMIR408-sgRNA02+sgRNA04-18
Allele-1:                 GAGGAGAGGAGACAGGGATGAGGCAGAGCATGGGCATGGGGCTATCAACAGATGTAG......55bp......TGGTGTTGTTGTTGCTCCCTC-C--TGCACTGCCTCTTCCCTGGCTCCCCTGCAC (+1  bp//-2 bp)
Allele-2:                 GAGGAGAGGAGACAGGGATGAGGCAGAGCATGGG----------------------------------------------------CCTGCACTGCCTCTTCCCTGGCTCCCCTGCAC (-99  bp)
#OsMIR408-sgRNA02+sgRNA04-19
Allele-1:                 GAGGAGAGGAGACAGGGATGAGGCAGAGCATGGG------GCTATCAACAGATGTAG......55bp......TGGTGTTGTTGTTGCTCCCTC-CC-TGCACTGCCTCTTCCCTGGCTCCCCTGCAC (-5  bp//-1 bp)
Allele-2:                 GAGGAGAGGAGACAGGGATGAGGCAGAGCATGGG--------TATCAACAGATGTAG......55bp......TGGTGTTGTTGTTGCTCCCTCCCCGTGCACTGCCTCTTCCCTGGCTCCCCTGCAC (-7  bp//+1 bp)
#OsMIR408-sgRNA02+sgRNA04-20
Allele-1:                 GAGGAGAGGAGACAGGGATGAGGCAGAGCATGGG------GCTATCAACAGATGTAG......55bp......TGGTGTTGTTGTTGCTCCCT-----TGCACTGCCTCTTCCCTGGCTCCCCTGCAC (-5  bp//-4 bp)
Allele-2:                 GAGGAGAGGAGACAGGGATGAGGCAGAGCATGGG----------------------......20bp......TGGTGTTGTTGTTGCTCCCTCCGCCTGCACTGCCTCTTCCCTGGCTCCCCTGCAC (-57 bp//+1 bp)
```

Supplementary Fig 17. Genotyping rice T0 lines for deletions at *OsMIR528* Allele

pZmUbi1::Cas9+OsU6::OsMIR*528*-sgRNA01+sgRNA03 (pZJP026)

```
Reference:    GCAGCAGCAGTGGAAGGGGCATGCAGAGG--AGCAGGAGATTCAGTTTGAAGCTGGACTTCACTTTTGCCTCTCTCTCCTGTGC-TTGCCTCTTCCATTCCTGCTGCTAGGCTGTTCT
#OsMIR528-sgRNA01+sgRNA03-01
Allele-1:     GCAGCAGCAGTGGAAGGGGCATGCAGAG---AGCAGGAGATTCAGTTTGAAGCTGGACTTCACTTTTGCCTCTCTCTCCTGTGCCTTGCCTCTTCCATTCCTGCTGCTAGGCTGTTCT (-1 bp//+1 bp)
Allele-2:     GCAGCAGCAGTGGAAGGGGCATGCAGAGG--AGCAGGAGATTCAGTTTGAAGCTGGACTTCACTTTTGCCTCTCTCTCCTGTGC-TTGCCTCTTCCATTCCTGCTGCTAGGCTGTTCT (WT//WT)
#OsMIR528-sgRNA01+sgRNA03-02
Allele-1:     GCAGCAGCAGTGGAAGGGGCATGCAGAGG--AGCAGGAGATTCAGTTTGAAGCTGGACTTCACTTTTGCCTCTCTCTCCTGTGC-TTGCCTCTTCCATTCCTGCTGCTAGGCTGTTCT (WT//WT)
Allele-2:     GCAGCAGCAGTGGAAGGGGCATGCAGAGG--AGCAGGAGATTCAGTTTGAAGCTGGACTTCACTTTTGCCTCTCTCTCCTGTGC-TTGCCTCTTCCATTCCTGCTGCTAGGCTGTTCT (WT//WT)
#OsMIR528-sgRNA01+sgRNA03-03
Allele-1:     GCAGCAGCAGTGGAAGGGGCATGCAGAGG------------------------------------------------------GC-TTGCCTCTTCCATTCCTGCTGCTAGGCTGTTCT (-51 bp)
Allele-2:     GCAGCAGCAGTGGAAGGGGCATGCAGAGGT-AGCAGGAGATTCAGTTTGAAGCTGGACTTCACTTTTGCCTCTCTCTCCTGTGC-TTGCCTCTTCCATTCCTGCTGCTAGGCTGTTCT (-1 bp//WT)
#OsMIR528-sgRNA01+sgRNA03-04
Allele-1:     GCA------------------------------------------------------------------------------------------------------------------- (-164 bp)
Allele-2:     GCAGCAGCAGTGGAAGGGGCATGC-----T-AGCAGGAGATTCAGTTTGAAGCTGGACTTCACTTTTGCCTCTCTCTCCTGTGACTTGCCTCTTCCATTCCTGCTGCTAGGCTGTTCT (-5 bp, △1 bp//+1 bp)
#OsMIR528-sgRNA01+sgRNA03-05
Allele-1:     GCAGCAGCAGTGGAAGGGGCATGC--AGG--AGCAGGAGATTCAGTTTGAAGCTGGACTTCACTTTTGCCTCTCTCTCCTGTGACTTGCCTCTTCCATTCCTGCTGCTAGGCTGTTCT (-2 bp//+1 bp)
Allele-2:     GCAGCAGCAGTGGAAGGGGCATGCA-A----A-CAGGAGATTCAGTTTGAAGCTGGACTTCACTTTTGCCTCTCTCTCCTGTGTCTTGCCTCTTCCATTCCTGCTGCTAGGCTGTTCT (-4 bp//+1 bp)
#OsMIR528-sgRNA01+sgRNA03-06
Allele-1:     GCAGCAGCAGTGGAAGGGGCATGC--AGG--AGCAGGAGATTCAGTTTGAAGCTGGACTTCACTTTTGCCTCTCTCTCCTGTGACTTGCCTCTTCCATTCCTGCTGCTAGGCTGTTCT (-2 bp//+1 bp)
Allele-2:     GCAGCAGCAGTGGAAGGGGCATGCA-A----A-CAGGAGATTCAGTTTGAAGCTGGACTTCACTTTTGCCTCTCTCTCCTGTGTCTTGCCTCTTCCATTCCTGCTGCTAGGCTGTTCT (-4 bp//+1 bp)
#OsMIR528-sgRNA01+sgRNA03-07
Allele-1:     GCAGCAGCAGTGGAAGGGGCATGCAGAGG--CACAGGAGAGAGAGGCAAAAGTGAAGTCCAGCTTCAAACTGAATCTCCTGCT-CTTGCCTCTTCCATTCCTGCTGCTAGGCTGTTCT (-52 bp inversion)
Allele-2:     GCAGCAGCAGTGGAAGGGGCATGCAGAGGT-AGCAGGAGATTCAGTTTGAAGCTGGACTTCACTTTTGCCTCTCTCTCCTGTG-----CCTCTTCCATTCCTGCTGCTAGGCTGTTCT (+1 bp//+4 bp)
#OsMIR528-sgRNA01+sgRNA03-08
Allele-1:     GCAGCAGCAGTGGAAGGGGCATGCAGAGG------------------------------------------------------CTTGCCTCTTCCATTCCTGCTGCTAGGCTGTTCT (52 bp deletion)
Allele-2:     GCAGCAGCAGTGGAAGGGGCATGCAG-----AGCAGGAGATTCAGTTTGAAGCTGGACTTCACTTTTGCCTCTCTCTCCTGTGACTTGCCTCTTCCATTCCTGCTGCTAGGCTGTTCT (-3 bp//+1 bp)
#OsMIR528-sgRNA01+sgRNA03-09
Allele-1:     GCAGCAGCAGTGGAAGGGGCATGCAGAGG--AGCAGGAGATTCAGTTTGAAGCTGGACTTCACTTTTGCCTCTCTCTCCTGTGC-TTGCCTCTTCCATTCCTGCTGCTAGGCTGTTCT (WT//WT)
Allele-2:     GCAGCAGCAGTGGAAGGGGCATGCAGAGG--AGCAGGAGATTCAGTTTGAAGCTGGACTTCACTTTTGCCTCTCTCTCCTGTGC-TTGCCTCTTCCATTCCTGCTGCTAGGCTGTTCT (WT//WT)
#OsMIR528-sgRNA01+sgRNA03-10
Allele-1:     GCAGCAGCAGTGGAAGGGGCATGCAGAGG--AGCAGGAGATTCAGTTTGAAGCTGGACTTCACTTTTGCCTCTCTCTCCTGTGC-TTGCCTCTTCCATTCCTGCTGCTAGGCTGTTCT (WT//WT)
Allele-2:     GCAGCAGCAGTGGAAGGGGCATGCAGAGG--AGCAGGAGATTCAGTTTGAAGCTGGACTTCACTTTTGCCTCTCTCTCCTGTGC-TTGCCTCTTCCATTCCTGCTGCTAGGCTGTTCT (WT//WT)
#OsMIR528-sgRNA01+sgRNA03-11
Allele-1:     GCAGCAGCAGTGGAAGG--------------------AGATTCAGTTTGAAGCTGGACTTCACTTTTGCCTCTCTCTCCTGTG-CT-GCCTCTTCCATTCCTGCTGCTAGGCTGTTCT (-18 bp//-1 bp)
Allele-2:     GCAGCAGCAGTGGAAGGGGCATGCAGAG---AGCAGGAGATTCAGTTTGAAGCTGGACTTCACTTTTGCCTCTCTCTCCTGTGCCTTGCCTCTTCCATTCCTGCTGCTAGGCTGTTCT (-1 bp//+1 bp)
#OsMIR528-sgRNA01+sgRNA03-12
Allele-1:     GCAGCAGCAGTGGAAGGGGCATGCAGAG---AGCAGGAGATTCAGTTTGAAGCTGGACTTCACTTTTGCCTCTCTCTCCTGTGA---GCCTCTTCCATTCCTGCTGCTAGGCTGTTCT (-1 bp//-3 bp, △1 bp)
Allele-2:     GCAGCAGCAGTGGAAGGGGCATGCAGAG---AGCAGGAGATTCAGTTTGAAGCTGGACTTCACTTTTGCCTCTCTCTCCTGTGA---GCCTCTTCCATTCCTGCTGCTAGGCTGTTCT (-1 bp//-3 bp, △1 bp)
#OsMIR528-sgRNA01+sgRNA03-13
Allele-1:     GCAGCAGCAGTGGAAGGGGCATGCAGAGG------------------------------------------------------CTTGCCTCTTCCATTCCTGCTGCTAGGCTGTTCT (-52 bp)
Allele-2:     GCAGCAGCAGTGGAAGGGGCATGCAGAGG------------------------------------------------------CTTGCCTCTTCCATTCCTGCTGCTAGGCTGTTCT (-52 bp)
#OsMIR528-sgRNA01+sgRNA03-14
Allele-1:     GCAGCAGCAGTGGAAGGGGCATGCAGAGGC-AGCAGGAGATTCAGTTTGAAGCTGGACTTCACTTTTGCCTCTCTCTCCTGTGACTTGCCTCTTCCATTCCTGCTGCTAGGCTGTTCT (+1 bp//+1 bp)
Allele-2:     GCAGCAGCAGTGGAAGGGGCATGCAGAGGATAGCAGGAGATTCAGTTTGAAGCTGGACTTCACTTTTGCCTCTCTCTCCTGTGACTTGCCTCTTCCATTCCTGCTGCTAGGCTGTTCT (+2 bp//+1 bp)
#OsMIR528-sgRNA01+sgRNA03-15
Allele-1:     GCAGCAGCAGTGGAAGGGGCATGCAGAGGC-AGCAGGAGATTCAGTTTGAAGCTGGACTTCACTTTTGCCTCTCTCTCCTGTGACTTGCCTCTTCCATTCCTGCTGCTAGGCTGTTCT (+1 bp//+1 bp)
Allele-2:     GCAGCAGCAGTGGAAGGGGCATGCAGAGGATAGCAGGAGATTCAGTTTGAAGCTGGACTTCACTTTTGCCTCTCTCTCCTGTGACTTGCCTCTTCCATTCCTGCTGCTAGGCTGTTCT (+2 bp//+1 bp)
#OsMIR528-sgRNA01+sgRNA03-16
Allele-1:     GCAGCAGCAGTGGAAGGGGCATGCAGAGG------------------------------------------------------CTTGCCTCTTCCATTCCTGCTGCTAGGCTGTTCT (-52 bp)
Allele-2:     GCAGCAGCAGTGGAAGGGGCATGCAGAGG------------------------------------------------------CTTGCCTCTTCCATTCCTGCTGCTAGGCTGTTCT (-52 bp)
#OsMIR528-sgRNA01+sgRNA03-17
Allele-1:     GCAGC--------------------------AGCAGGAGATTCAGTTTGAAGCTGGACTTCACTTTTGCCTCTCTCTCCTGTG--TTGCCTCTTCCATTCCTGCTGCTAGGCTGTTCT (-24 bp//-1 bp)
Allele-2:     GCAGCAGCAGTGGAAGGGGCAT----------GCAGGAGATTCAGTTTGAAGCTGGACTTCACTTTTGCCTCTCTCTCCTGTG----GCCTCTTCCATTCCTGCTGCTAGGCTGTTCT (-8 bp//-3 bp)
#OsMIR528-sgRNA01+sgRNA03-18
Allele-1:     GCAGCAGCAGTGGAAGGGGCATGCAGAG-C-A-C--GAGATTCAGTTTGAAGCTGGACTTCACTTTTGCCTCTCTCTCCTGTGCCTTGCCTCTTCCATTCCTGCTGCTAGGCTGTTCT (-4 bp, +1 bp//+1 bp)
Allele-2:     GCAGCAGCAGTGGAAGGGGCAT-----GG--AGCAGGAGATTCAGTTTGAAGCTGGACTTCACTTTTGCCTCTCTCTCCTGTG----GCCTCTTCCATTCCTGCTGCTAGGCTGTTCT (-5 bp//-3 bp)
#OsMIR528-sgRNA01+sgRNA03-19
Allele-1:     GCAGCAGCAGTGGAAGGGGCATGCAGAG--------GAGATTCAGTTTGAAGCTGGACTTCACTTTTGCCTCTCTCTCCTG-----TGCCTCTTCCATTCCTGCTGCTAGGCTGTTCT (-6bp//-4 bp)
Allele-2:     GCAGCAGCAGTGGAAGGGGCATGCAGAG---AGC---AGATTCAGTTTGAAGCTGGACTTCACTTTTGCCTCTCTCTCCTGTG---TGCCTCTTCCATTCCTGCTGCTAGGCTGTTCT (-4 bp//-2 bp)
#OsMIR528-sgRNA01+sgRNA03-20
Allele-1:     GCAGCAGCAGTGGAAGGGGCATGCAGAG--------GAGATTCAGTTTGAAGCTGGACTTCACTTTTGCCTCTCTCTCCTG-----TGCCTCTTCCATTCCTGCTGCTAGGCTGTTCT (-6bp//-4 bp)
Allele-2:     GCAGCAGCAGTGGAAGGGGCATGCAGAG---AGC---AGATTCAGTTTGAAGCTGGACTTCACTTTTGCCTCTCTCTCCTGTG---TGCCTCTTCCATTCCTGCTGCTAGGCTGTTCT (-4 bp//-2 bp)
#OsMIR528-sgRNA01+sgRNA03-21
Allele-1:     GCAGCAGCAGTGGAAGGGGCATGCAGAG---AGCAGGAGATTCAGTTTGAAGCTGGACTTCACTTTTGCCTCTCTCTCCTGTGCCTTGCCTCTTCCATTCCTGCTGCTAGGCTGTTCT (-1 bp//+1 bp)
Allele-2:     GCAGCAGCAGTGGAAGGGGCAT----------GCAGGAGATTCAGTTTGAAGCTGGACTTCACTTTTGCCTCTCTCTCCTGTGCCTTGCCTCTTCCATTCCTGCTGCTAGGCTGTTCT (-8 bp//+1 bp)
#OsMIR528-sgRNA01+sgRNA03-22
Allele-1:     GCAGCAGCAGTGGAAGGGGCATGCAGAGGT-AGCAGGAGATTCAGTTTGAAGCTGGACTTCACTTTTGCCTCTCTCTCCTGTGACTTGCCTCTTCCATTCCTGCTGCTAGGCTGTTCT (+1 bp//+1 bp)
Allele-2:     GCAGCAGCAGTGGAAGGGGCATGCAG---C-AGCAGGAGATTCAGTTTGAAGCTGGACTTCACTTTTGCCTCTCTCTCCTGTGACTTGCCTCTTCCATTCCTGCTGCTAGGCTGTTCT (-3bp, +1 bp//+1 bp)
```

Table S1. Oligos used in this study

Tested site	Oligo name	Oligo sequence	Note
OsMIR408-sgRNA03	OsMIR408-sgRNA-F3	GTGTCCCCTGCACTGCCTCTTCCC	vectors construction
	OsMIR408-sgRNA-R3	AAACGGGAAGAGGCAGTGCAGGGG	
OsMIR528-sgRNA01	OsMIR528-sgRNA-F1	GTGTGAAGGGGCATGCAGAGGAGC	vectors construction
	OsMIR528-sgRNA-R1	AAACGCTCCTCTGCATGCCCCTTC	
OsMIR528-sgRNA02	OsMIR528-sgRNA-F2	GTGTGCAGTGGAAGGGGCATGCAG	vectors construction
	OsMIR528-sgRNA-R2	AAACCTGCATGCCCCTTCCACTGC	
OsMIR528-sgRNA03	OsMIR528-sgRNA-F3	GTGTGGAATGGAAGAGGCAAGCAC	vectors construction
	OsMIR528-sgRNA-R3	AAACGTGCTTGCCTCTTCCATTCC	
OsMIR815-sgRNA01	OsMIR815-sgRNA-F	GTGTGAGAAGGGGATTGAGGAGAT	vectors construction
	OsMIR815-sgRNA-R	AAACATCTCCTCAATCCCCTTCTC	
OsMIR820-sgRNA01	OsMIR820-sgRNA-F	GTGTGTCGGCCTCGTGGATGGACC	vectors construction
	OsMIR820-sgRNA-R	AAACGGTCCATCCACGAGGCCGAC	
OsMIR408-sgRNA02+sgRNA04	miR408-gRNA-F1	CACCGGTCTCAGTGTGGATGAGGCA GAGCATGGGAGTTTTAGAGCTAGAAATA	vectors construction
	miR408-gRNA-R1	CGATGGTCTCCAAACTCCCCTGCAC TGCCTCTTCCACACAAGCGACAGCG C	
OsMIR528-sgRNA01+sgRNA03	miR528-gRNA-F1	CACCGGTCTCAGTGTGAAGGGGCAT GCAGAGGAGCGTTTTAGAGCTAGAAATA	vectors construction
	miR528-gRNA-R1	CGATGGTCTCCAAACGTGCTTGCCTC TTCCATTCCACACA AGCGACAGCG C	
-	General-P1R	CGCCAATATATCCTGTCAAA	vectors construction
-	General-P2F	TTTGACAGGATATATTGGCGAGGATCCGCGGATCATG	vectors construction
pri-miR528	OsMIR528-F1	CACCAATGGATGCATCAGCAG	NHEJ event detection
	OsMIR528-SphI-F2	GCGTTTGCTCCAACGCTTCTG	
	OsMIR528-R	TGAGAGTTTGGTGCAATAACAG	
pri-miR408	OsMIR408-F	ATCTTGACGATGATGGCGTTG	NHEJ event detection
	OsMIR408-R	AGAGAGAGAGAGAGAGGTGTG	
pri-miR815a	OsMIR815a-SSCP-F	CCTGATTGACCTGTTTGCTGT	NHEJ event detection
	OsMIR815a-SSCP-R	TTCAAACTACTAAACGGTGC GT	
pri-miR815b	OsMIR815b-SSCP-F	GTGTGTAATTAGCTGCGTTCT	NHEJ event detection
	OsMIR815b-SSCP-R	GTACATTAAGGTATCGTTCGTT	
pri-miR815c	OsMIR815c-SSCP-F	GAGCCCATTTATCTCTCACAC	NHEJ event detection
	OsMIR815c-SSCP-R	CACACGCACGCTTCCCAAAC	
pri-miR820a	OsMIR820a-SSCP-F	ATGACATGCGTTGGTCATCCG	NHEJ event detection
	OsMIR820a-SSCP-R	GGCTAGCTCGTGTGTTCAAAG	
pri-miR820b	OsMIR820b-SSCP-F	ATGACATGCGTTGGTCATCCA	NHEJ event detection
	OsMIR820b,c-SSCP-R	GGCTAGCTCGTGTGTTCAAAT	
pri-miR820c	OsMIR820c-SSCP-F	ATGACATGCGTTGGTCATTCG	NHEJ event detection
OsMIR408-sgRNA03-OT1	miR408-sgRNA03-OT1-F	CTCCACCGGT GCTGCTCCT	off-targeting test
	miR408-sgRNA03-OT1-R	GAAGGTCGACAAGGCTTGCAT	
OsMIR408-sgRNA03-OT2	miR408-sgRNA03-OT2-F	CGATCTCTCCGAACGAACACA	off-targeting test
	miR408-sgRNA03-OT2-R	GATCACTCACCAAGGACGTCA	
OsMIR408-sgRNA03-OT3	miR408-sgRNA03-OT3-F	CGACATCGCCAATCTGCACT	off-targeting test
	miR408-sgRNA03-OT3-R	GAGGTGGAGTAGCAATGAGGT	
OsMIR408-sgRNA03-OT4	miR408-sgRNA03-OT4-F	GCAACAAGACAAGAGTAATCT	off-targeting test
	miR408-sgRNA03-OT4-R	GACTAGCGATGGTGGATTTAG	
	miR408-sgRNA03-OT5-F	TCGCCCACGTAGTACGTCTT	
	miR408-sgRNA03-OT5-R	CACCACACGAGTCAGAAGACA	

Table S1. Oligos used in this study (continued)

Tested site	Oligo name	Oligo sequence	Note
OsMIR528-sgRNA01-OT1	miR528-sgRNA01-OT1-F	CGAGTTCATCTGCCCCAAGT	off-targeting test
	miR528-sgRNA01-OT1-R	GAGGCCATGGGGCACATTGA	
OsMIR528-sgRNA01-OT2	miR528-sgRNA01-OT2-F	CGTCGATCCCATCAGCACCA	off-targeting test
	miR528-sgRNA01-OT2-R	GCCCATGGATCTCGCATCCT	
OsMIR528-sgRNA01-OT3	miR528-sgRNA01-OT3-F	CGTCCTGTCTATGGTAGGTA	off-targeting test
	miR528-sgRNA01-OT3-R	GCCAGGTTCTCTTTGCATGA	
OsMIR528-sgRNA01-OT4	miR528-sgRNA01-OT4-F	GTAGGCCCCACATCCTTTCT	off-targeting test
	miR528-sgRNA01-OT4-R	CGTTGCTGGAGGACAAGAAC	
OsMIR528-sgRNA01-OT5	miR528-sgRNA01-OT5-F	CGGTAAGACGGACCATCAAGA	off-targeting test
	miR528-sgRNA01-OT5-R	CCCTTCTCGCACACACTATAG	
OsMIR528-sgRNA02-OT1	miR528-sgRNA02-OT1-F	GACTCCAGAGTAACAATAGCA	off-targeting test
	miR528-sgRNA02-OT1-R	CAAGAGGAGAGACAAATCATG	
OsMIR528-sgRNA02-OT2	miR528-sgRNA02-OT2-F	TCCATCCAACTACACGCTGA	off-targeting test
	miR528-sgRNA02-OT2-R	GGACTAAGTAAAAGCCAACGA	
OsMIR528-sgRNA02-OT3	miR528-sgRNA02-OT3-F	GCAACCGGATCTGGCGTAAC	off-targeting test
	miR528-sgRNA02-OT3-R	AATGTGTCCGGCTTGCTTATG	
OsMIR528-sgRNA02-OT4	miR528-sgRNA02-OT4-F	CGACGCCGGTGCGGGAGAAG	off-targeting test
	miR528-sgRNA02-OT4-R	GTGCTCCTCTCCTCTTCACGA	
OsMIR528-sgRNA02-OT5	miR528-sgRNA02-OT5-F	GCACCACATCCATTGCCACT	off-targeting test
	miR528-sgRNA02-OT5-R	CCATCGTTGGCAATATGTGTC	
OsMIR528-sgRNA03-OT1	miR528-sgRNA03-OT1-F	GTCAAGGAGGAGGAGGGAGA	off-targeting test
	miR528-sgRNA03-OT1-R	CCGATTCCCTTGGGTCCACA	
OsMIR528-sgRNA03-OT2	miR528-sgRNA03-OT2-F	CGTGGGCCTGCGGATCTGCT	off-targeting test
	miR528-sgRNA03-OT2-R	CCTTAGAGCCACCGCGAACA	
OsMIR528-sgRNA03-OT3	miR528-sgRNA03-OT3-F	CGGACACAAGTCCATGATCCT	off-targeting test
	miR528-sgRNA03-OT3-R	CCTCCTCCTCCTGCTGCTAC	
OsMIR528-sgRNA03-OT4	miR528-sgRNA03-OT4-F	CGACTTCTGAACTACTGACCT	off-targeting test
	miR528-sgRNA03-OT4-R	AGCATGGTGTCCAACTGATTC	
OsMIR528-sgRNA03-OT5	miR528-sgRNA03-OT5-F	ACGTGCGGAACAAGATCAGCA	off-targeting test
	miR528-sgRNA03-OT5-R	GGCCTCCAGCTGCTTAATCA	
OsMIR815-sgRNA01-OT1	miR815-sgRNA01-OT1-F	GTAGCACCAGAGGTCGTACT	off-targeting test
	miR815-sgRNA01-OT1-R	GGGAGTGAGAATGCACATCGT	
OsMIR815-sgRNA01-OT2	miR815-sgRNA01-OT2-F	CCTCTCCTGTCTATCACAACT	off-targeting test
	miR815-sgRNA01-OT2-R	GCACGCACGCTTCCTAAACT	
OsMIR815-sgRNA01-OT3	miR815-sgRNA01-OT3-F	CCTCCGGCGAGAACCCTAGA	off-targeting test
	miR815-sgRNA01-OT3-R	CCTCCTCCTTGCGAATCCAA	
OsMIR815-sgRNA01-OT4	miR815-sgRNA01-OT4-F	GACCCCTAGTCGTTTCTCAAG	off-targeting test
	miR815-sgRNA01-OT4-R	CGCACGCTTTTCAAACTACT	
OsMIR815-sgRNA01-OT5	miR815-sgRNA01-OT5-F	GCGGAAAACGAGTGACAATCT	off-targeting test
	miR815-sgRNA01-OT5-R	CCTATGTACTTAGCACATCGT	
OsMIR820-sgRNA01-OT1	miR820-sgRNA01-OT1-F	CATGGCTAGCTCGTGTGATCA	off-targeting test
	miR820-sgRNA01-OT1-R	GCTAGCACGTCTATTGAAAGA	
OsMIR820-sgRNA01-OT2	miR820-sgRNA01-OT2-F	CAGGCAGCGAAACCTTGTATG	off-targeting test
	miR820-sgRNA01-OT2-R	CATGCCGCTATAATACGAAGT	
OsMIR820-sgRNA01-OT3	miR820-sgRNA01-OT3-F	GAGTGGGACACAGACGGTGA	off-targeting test
	miR820-sgRNA01-OT3-R	CCTTCCCATTTGCATCCTTCA	
OsMIR820-sgRNA01-OT4	miR820-sgRNA01-OT4-F	ATGTGCTAGCACGTCTATTGA	off-targeting test
	miR820-sgRNA01-OT4-R	CTAACTCCTAGCTGAGTCGT	
OsMIR820-sgRNA01-OT5	miR820-sgRNA01-OT5-F	GAAGCGATGGACATCACCTTG	off-targeting test
	miR820-sgRNA01-OT5-R	GTCCTCCTGCTGACGAACAG	

Table S1. Oligos used in this study (continued)

Tested site	Oligo name	Oligo sequence	Note
OsMIR408	OsMIR408	CTGCACTGCCTCTTCCCTGGC	Real-time PCR
OsMIR528	OsMIR528	TGGAAGGGGCATGCAGAGGAG	Real-time PCR
OsMIR168a	OsMIR168a	TCGCTTGGTGCAGATCGGGAC	Real-time PCR
OsMIR396e	OsMIR396e	TCCACAGGCTTTCTTGAACTG	Real-time PCR
OsMIR156	OsMIR156	TGACAGAAGAGAGTGAGCAC	Real-time PCR
OsMIR167	OsMIR167	TGAAGCTGCCAGCATGATCT	Real-time PCR
OsMIR398	OsMIR398	TGTGTTCTCAGGTCGCCCCT	Real-time PCR
OsMIR159	OsMIR159	TTGGATTGAAGGGAGCTC	Real-time PCR
OsMIR397	OsMIR397	ATTGAGTGCAGCGTTGATG	Real-time PCR
OsMIR162a	OsMIR162a	TCGATAAACCTCTGCATCCAG	Real-time PCR
OsTubulin	OsTubulin-F	GGACTCTCAAGCTGACCACAC	Real-time PCR
	OsTubulin-R	GCGAATCCGACCATGAAGAAG	
Os03g0129400	Os03g0129400-F	CAGTTTGAAGCTGGACTTCACT	Real-time PCR
	Os03g0129400-R	GCTGCTGATGCTGATGCCATG	
Os06g0567900	Os06g0567900-F	GCCCATTCCAGTTAAATGCCA	Real-time PCR
	Os06g0567900-R	TGAGCATGCATATGACCAAACA	
Os08g0137400	Os08g0137400-F	CTCTCTGATTGTTCTCTTCTG	Real-time PCR
	Os08g0137400-R	GAGCGACCCACTCTTTGTAG	
Os06g0154200	Os06g0154200-F	CCATTTGCTGATTCGTTCTCTG	Real-time PCR
	Os06g0154200-R	CTCGCATTATCTTAACTAGCTG	
Os07g0570550	Os07g0570550-F	GCCGATCGCCACCTGGTCGA	Real-time PCR
	Os07g0570550-R	ACGGGTGCCTCGGAGCCGGA	
Os03g0259100	Os03g0259100-F	GCTCGCGTAGTCGCGTACTC	RT-PCR
	Os03g0259100-R	CGGACGGGTCGTAGCTGAAC	

Table S2. Heredity test at targeted *OsMIR528* site in T1 generation

T0 seedling	T0 mutation	T1 number	T1 mutation	X^2-Test
WT	AGCAGCAGTGGAAGGGGCATGCAGAGG-AGCAGGAGATTCAGTTTGAAGCT<br>AGCAGCAGTGGAAGGGGCATGCAGAGG-AGCAGGAGATTCAGTTTGAAGCT	-	-	-
OsMIR528-<br>sgRNA01-01	AGCAGCAGTGGAAGGGGCA---------AGCAGGAGATTCAGTTTGAAGCT (-8 bp)<br>AGCAGCAGTGGAAGGGGCATGCAGA--TAGCAGGAGATTCAGTTTGAAGCT (-2/+1 bp)	42	9 : -8 bp//-8 bp<br>26: -8 bp//-2/+1 bp<br>7 : -2/+1 bp//-2/+1 bp	$X^2=2.00<5.99_{p=0.05,df=2}$
OsMIR528-<br>sgRNA01-04	AGCAGCAGTGGAAGGGGCATGCAGAG--AGCAGGAGATTCAGTTTGAAGCT (-1 bp)<br>AGCAGCAGTGGAAGGGGCATGCAGAG----CAGGAGATTCAGTTTGAAGCT (-3 bp)	47	8 : -1 bp//-1 bp<br>25: -1 bp//-3 bp<br>14: -3 bp//-3 bp	$X^2=1.72<5.99_{p=0.05,df=2}$
OsMIR528-<br>sgRNA01-08	AGCAGCAGTGGAAGGGGCATGCAG------------------TTTGAAGCT (-17 bp)<br>AGCAGCAGTGGAAGGGGCATGCAG----AGCAGGAGATTCAGTTTGAAGCT (-3 bp)	56	10: -17 bp//-17 bp<br>33: -17 bp//-3 bp<br>13: -3 bp//-3 bp	$X^2=2.11<5.99_{p=0.05,df=2}$
OsMIR528-<br>sgRNA01-09	AGCAGCAGTGGAAGGGGCATGCAG-----GCAGGAGATTCAGTTTGAAGCT (-4 bp)<br>AGCAGCAGTGGAAGGGGCATGCAGAGGGAGCAGGAGATTCAGTTTGAAGCT (+1 bp)	61	13: -4 bp//-4 bp<br>37: -4 bp//+1 bp<br>11: +1 bp//+1 bp	$X^2=2.90<5.99_{p=0.05,df=2}$
OsmiR528-<br>sgRNA01-10	AGCAGCAGTGGAAGGGGCATGCAGAGG--GCAGGAGATTCAGTTTGAAGCT (-1 bp)<br>AGCAGCAGTGGAAGGGGCATGCAGAGGTAGCAGGAGATTCAGTTTGAAGCT (+1 bp)	52	14: -1 bp//-1 bp<br>28: -1 bp//+1 bp<br>10: +1 bp//+1 bp	$X^2=0.92<5.99_{p=0.05,df=2}$
OsmiR528-<br>sgRNA01-11	AGCAGCAGTGGAAGGGGCATGCAGA---AGCAGGAGATTCAGTTTGAAGCT (-2 bp)<br>AGCAGCAGTGGAAGGGGCATGCAGA----GCAGGAGATTCAGTTTGAAGCT (-3 bp)	49	15: -2 bp//-2 bp<br>25: -2 bp//-3 bp<br>9 : -3 bp//-3 bp	$X^2=1.49<5.99_{p=0.05,df=2}$
OsMIR528-<br>sgRNA01-33	AGCAGCAGTGGAAGGGGCATGCAGAGGAAGCAGGAGATTCAGTTTGAAGCT (+1 bp)<br>AGCAGCAGTGGAAGGGGCATGCAGAGGAAGCAGGAGATTCAGTTTGAAGCT (+1 bp)	10	10: +1 bp//+1 bp	-

Table S3. Simultaneous targeting of homologous microRNAs in T0 rice lines

	miR815a		miR815b		miR815c	
	Allele 1	Allele 2	Allele 1	Allele 2	Allele 1	Allele 2
OsMIR815-sgRNA01-01	-6bp	-2bp	-1bp	-1bp	-10bp	-1bp
OsMIR815-sgRNA01-05	-6bp	-2bp	-1bp	-1bp	-10bp	-1bp
OsMIR815-sgRNA01-06	-8bp	wt	-6bp	wt	-3bp	wt
OsMIR815-sgRNA01-08	-2bp	-1bp	-1bp	wt	wt	wt
OsMIR815-sgRNA01-09	-2bp	-2bp	-3bp	-2bp	-2bp	wt
OsMIR815-sgRNA01-10	-5bp	-3bp	-3bp	wt	-2bp	wt
OsMIR815-sgRNA01-11	-5bp	-3bp	-3bp	wt	-2bp	wt
OsMIR815-sgRNA01-12	-5bp	-3bp	-3bp	wt	-2bp	wt
OsMIR815-sgRNA01-14	-3bp	wt	-2bp	△1bp	wt	wt
OsMIR815-sgRNA01-15	-2bp	-1bp	-1bp	wt	wt	wt
OsMIR815-sgRNA01-16	-2bp	-1bp	-1bp	wt	wt	wt
OsMIR815-sgRNA01-17	-3bp	wt	-2bp	△1bp	wt	wt
OsMIR815-sgRNA01-18	-5bp	wt	-7bp	-1bp	-2bp	wt
OsMIR815-sgRNA01-19	-5bp	-3bp	-3bp	wt	-2bp	wt
OsMIR815-sgRNA01-20	-5bp	wt	-5bp	+1bp	-8bp	-2bp
	miR820a		miR820b		miR820c	
	Allele 1	Allele 2	Allele 1	Allele 2	Allele 1	Allele 2
OsMIR820-sgRNA01-03-01	+1bp	+1bp	-2bp	+1bp	-2bp	-2bp
OsMIR820-sgRNA01-03-02	+1bp	+1bp	-2bp	+1bp	-2bp	-2bp
OsMIR820-sgRNA01-04-01	-2bp	+1bp	-2bp	+1bp	-3bp	-1bp
OsMIR820-sgRNA01-04-02	-2bp	+1bp	-2bp	+1bp	-3bp	-1bp
OsMIR820-sgRNA01-04-03	-2bp	+1bp	-2bp	+1bp	-3bp	-1bp
OsMIR820-sgRNA01-04-04	-2bp	+1bp	-2bp	+1bp	-3bp	-1bp
OsMIR820-sgRNA01-06	+1bp	-4bp	-1bp/△1bp	+1bp	+1bp	+1bp
OsMIR820-sgRNA01-07	+1bp	-4bp	-1bp/△1bp	+1bp	+1bp	+1bp
OsMIR820-sgRNA01-09-01	-7bp	wt	-4bp	-1/△1bp	-1bp	wt
OsMIR820-sgRNA01-09-02	-7bp	wt	-4bp	-1/△1bp	-1bp	wt
OsMIR820-sgRNA01-10-01	wt	wt	-1bp	+1bp	wt	wt
OsMIR820-sgRNA01-10-02	△1bp	wt	-1bp	wt	wt	wt
OsMIR820-sgRNA01-10-03	-28bp	-28bp	-4bp	-4bp	-2bp	-2bp
OsMIR820-sgRNA01-10-04	+1bp	+1bp	-4bp	+1bp	-2bp	-3bp

Table S4. Summary of off-targeting analysis with the sgRNAs used in this study

Targeted Allele	Putative off-target site	Putative off-target Allele	Sequence	Mismatched Bases	Off target frequency((5 plants were used to check)
OsMIR408-sgRNA03	-	-	CCCCTGCACTGCCTCTTCCCTGG	-	-
	OsMIR408-sgRNA03-OT1	chr8: 1233011-1233033	CCtCctCgCTGCCTCTTCCCCGG	4	NO
	OsMIR408-sgRNA03-OT2	chr3:8375706-83757295	gCgCTGCACTGCCTCTTCCCTGG	2	No
	OsMIR408-sgRNA03-OT3	chr1:12322606-12322628	tCCgTGCAtTGCCTCTTCCCTGG	3	NO
	OsMIR408-sgRNA03-OT4	chr6:5285902-5285924	CtCgTGgcCTGCCTCTTCCCTGG	4	NO
	OsMIR408-sgRNA03-OT5	chr6:8836865-8836887	CgCtTGCACTGCCTCTTCCCCGG	2	NO
OsMIR528-sgRNA01	-	-	GAAGGGGCATGCAGAGGAGCAGG		
	OsMIR528-sgRNA01-OT1	chr8:7482559-7482581	GAAGGGGgAgGgAGAGGAGCAGG	3	NO
	OsMIR528-sgRNA01-OT2	chr1:14339517-14339539	GcAGaGcgAaGCAGAGGAGCAGG	5	NO
	OsMIR528-sgRNA01-OT3	chr7:22221540-22221562	GAAGGGGaAgGtAGAGGAGCAGG	3	NO
	OsMIR528-sgRNA01-OT4	chr9:4458644-4458666	GAgGcGGCcgGCAGAGGAGCAGG	4	NO
	OsMIR528-sgRNA01-OT5	chr2:27197057-27197079	GcctGGcgATGCAGAGGAGCAGG	5	NO
OsMIR528-sgRNA02	-	-	GCAGTGGAAGGGGCATG CAGAGG		
	OsMIR528-sgRNA02-OT1	chr8:14543012-14543034	GgAcTatAgGGGGCATGCAGCGG	3	NO
	OsMIR528-sgRNA02-OT2	chr11:2288006-2288028	GCAGTGaAAacaGCATGCAGAGG	4	NO
	OsMIR528-sgRNA02-OT3	chr11:11432378-11432400	ctttTGGcAGGGGCATGCAGCGG	5	NO
	OsMIR528-sgRNA02-OT4	chr9:22113144-22113166	GCAGTGGcgGGGGCATGCgGAGG	3	NO
	OsMIR528-sgRNA02-OT5	chr10:10257115-10257137	GCgaTGGcAttGGCATGCAGAGG	5	NO
OsMIR528-sgRNA03	-	-	GGAATGGAAGAGGCAAGCACAGG		
	OsMIR528-sgRNA03-OT1	chr8:20962409-20962431	GGgAgGGAAGAaGCAAGCACTGG	3	NO
	OsMIR528-sgRNA03-OT2	chr3:247560-247582	GGtATGGAgGAGGCAAGCACAGG	2	NO
	OsMIR528-sgRNA03-OT3	chr1:13305784-13305806	GaAgaaGAAGAaGCAAGCACAGG	5	NO
	OsMIR528-sgRNA03-OT4	chr2:30739866-30739888	GGAtTGcAAtgGaCAAGCACAGG	5	NO
	OsMIR528-sgRNA03-OT5	chr6:2782983-2783005	tGAATGGAAGAGGCAAGCAgAGG	2	NO
OsMIR815-sgRNA01	-	-	GAGAAGGGGATTGAGGAGATTGG		
	OsMIR815-sgRNA01-OT1	chr8:8554551-8554573	GAGAAaGGGATTGAGGAGATTGG	1	NO
	OsMIR815-sgRNA01-OT2	chr3:11202885-11202907	GAGAgGGGGATTGAGGAGATTGG	1	NO
	OsMIR815-sgRNA01-OT3	chr12:14193155-14193177	aAGAcGGGacTTGAGGAGATAGG	4	NO
	OsMIR815-sgRNA01-OT4	chr1:269519-269541	GgGAAGGGGATTGAGGAGATTGG	1	NO
	OsMIR815-sgRNA01-OT5	chr7:28846194-28846216	GAGgAGGGGATTGAGGAGATTGG	1	NO
OsMIR820-sgRNA01	-	-	GTCGGCCTCGTGGATGGACCAGG		
	OsMIR820-sgRNA01-OT1	chr8:19260375-19260397	aTCGtCCTCGTGGATGGACCTGG	2	NO
	OsMIR820-sgRNA01-OT2	chr8:19260537-19260559	GTCGGCCTCGTGGATaGACCAGG	1	NO
	OsMIR820-sgRNA01-OT3	chr3:611840-611862	GgCaGCCTCGTGGAcGGACCAGG	3	NO
	OsMIR820-sgRNA01-OT4	chr12:18631539-18631561	GTCGGCCTtcTGGATGGACCAGG	2	NO
	OsMIR820-sgRNA01-OT5	chr1:28211899-28211921	GTCGatCTgGaGGATGGACCTGG	4	NO

Table S5. Summary of highly differentially regulated microRNAs in microRNA genome-edited lines by high-throughput sequencing

Osa-microRNA ID	OsMIR408-sgRNA03-19	OsMIR528-sgRNA01-15	OsMIR528-sgRNA02-08	Up or down regulated
OsMIR1320-3p	-	-3.90	-	down
OsMIR1428e-3p	3.97	3.34	3.62	up
OsMIR1431	3.31	-	4.05	up
OsMIR1432-3p	-	-4.59	-	down
OsMIR167a-3p	-	-4.95	-	down
OsMIR169b	-	-4.11	-	down
OsMIR169i-5p.2	-3.15	-3.34	-	down
OsMIR169r-5p	-	-3.90	-	down
OsMIR172d-5p	-	-	-3.01	down
OsMIR1846a-5p	-	-3.37	-3.74	down
OsMIR1846a-3p	-	-	-5.15	down
OsMIR1846b-3p	-	-	-5.15	down
OsMIR1846b-5p	-	-3.37	-3.74	down
OsMIR1846c-5p	-	-3.37	-3.74	down
OsMIR1856	-	-	-3.89	down
OsMIR1861b	-	-3.46	-5.65	down
OsMIR1861e	-3.93	-3.37	-6.32	down
OsMIR1861f	-	-3.46	-5.65	down
OsMIR1861h	-	-	-3.34	down
OsMIR1861i	-	-3.46	-5.65	down
OsMIR1861j	-	-	-3.34	down
OsMIR1861k	-3.93	-3.37	-6.32	down
OsMIR1861l	-	-3.46	-5.65	down
OsMIR1861m	-3.93	-3.37	-6.32	down
OsMIR1881	-	-	4.00	up
OsMIR2090	6.03	3.17	4.81	up
OsMIR3979-5p	-	-25.89	-	down
OsMIR3979-3p	-	-26.31	-	down
OsMIR397a	7.67	7.02	7.77	up
OsMIR397b	7.39	7.51	7.16	up
OsMIR398a		3.07	-	up
OsMIR398b	5.85	7.24	6.90	up
OsMIR399a	-	-3.11	-	down
OsMIR399b	-	-3.11	-	down
OsMIR399c	-	-3.11	-	down
OsMIR408-5p	**	8.79	8.44	down/up/up
OsMIR408-3p	-3.39	9.01	9.03	down/up/up
OsMIR528-5p	6.57	-6.35	-6.14	up/down/down
OsMIR528-3p	5.96	-3.11	5.06	up/down/up
OsMIR5340	-7.09	-5.80	-30.91	down
OsMIR5542	3.93	-	3.19	up
OsMIR6249a	-27.89	-27.89	-27.89	down
OsMIR6249b	-27.89	-27.89	-27.89	down
OsMIR812k	-	-	-3.10	down
OsMIR812l	-	-	-3.10	down
OsMIR812m	-	-	-3.10	down

** Data are shown as normalized, log2-transformed fold-change of expression.
